# Ultrafast coherent motion and helix rearrangement of homodimeric hemoglobin visualized with femtosecond X-ray solution scattering

**DOI:** 10.1038/s41467-021-23947-7

**Published:** 2021-06-16

**Authors:** Yunbeom Lee, Jong Goo Kim, Sang Jin Lee, Srinivasan Muniyappan, Tae Wu Kim, Hosung Ki, Hanui Kim, Junbeom Jo, So Ri Yun, Hyosub Lee, Kyung Won Lee, Seong Ok Kim, Marco Cammarata, Hyotcherl Ihee

**Affiliations:** 1 Department of Chemistry and KI for the BioCentury, Korea Advanced Institute of Science and Technology (KAIST), Daejeon, Republic of Korea; 2 Center for Nanomaterials and Chemical Reactions, Institute for Basic Science (IBS), Daejeon, Republic of Korea; 3 European Synchrotron Radiation Facility, Grenoble, France

**Keywords:** SAXS, Photobiology, Reaction kinetics and dynamics

## Abstract

Ultrafast motion of molecules, particularly the coherent motion, has been intensively investigated as a key factor guiding the reaction pathways. Recently, X-ray free-electron lasers (XFELs) have been utilized to elucidate the ultrafast motion of molecules. However, the studies on proteins using XFELs have been typically limited to the crystalline phase, and proteins in solution have rarely been investigated. Here we applied femtosecond time-resolved X-ray solution scattering (fs-TRXSS) and a structure refinement method to visualize the ultrafast motion of a protein. We succeeded in revealing detailed ultrafast structural changes of homodimeric hemoglobin involving the coherent motion. In addition to the motion of the protein itself, the time-dependent change of electron density of the hydration shell was tracked. Besides, the analysis on the fs-TRXSS data of myoglobin allows for observing the effect of the oligomeric state on the ultrafast coherent motion.

## Abstract (web summary)

Femtosecond time-resolved X-ray solution scattering (fs-TRXSS) measurements provide information on the structural dynamics of proteins in solution. Here, the authors present a structure refinement method for the analysis of fs-TRXSS data and use it to characterise the ultrafast structural changes of homodimeric haemoglobin.

## Introduction

**Fig. 1:**
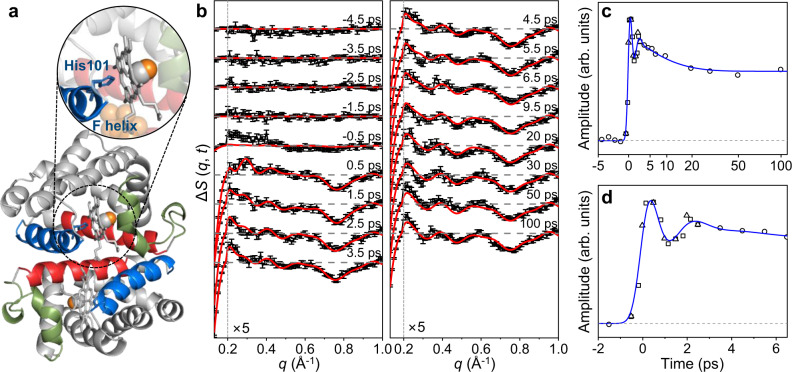
TRXSS data of HbI and temporal oscillation of the signal. **a** A crystal structure of HbI(CO)_2_ (bottom) and an enlarged view of the heme group and the His101 residue (top) (PDB entry: 3sdh). The C helix with the CD loop, the E helix, and the F helix are indicated with green, red, and blue colors, respectively. The CO ligand molecules are indicated with spheres. The photodissociation of a ligand molecule that is bound to a heme group induces the structural transition of the heme and the overall protein, called the R-T transition. **b** The experimental femtosecond TRXSS data (black) and calculated curves (red) from −4.5 to 100 ps. The calculated curve at each time delay shown in red was generated by taking a linear combination of the theoretical difference scattering curves of the reaction intermediates (I_0_, I_1_, I_0_^U^, and I_0_^D^) obtained from the structural refinement. For each species, the scattering curves of the candidate structures obtained from structure refinement were averaged to generate the corresponding theoretical difference scattering curve (see the Supplementary Information for detailed information). The coefficients for the linear combination were determined by the concentrations of the reaction intermediates at each time delay. For clarity, the high *q* region with *q* ≥ 0.2 Å^−1^ is scaled up by 5. **c**, **d** The first rSVs multiplied by their singular values obtained from the SVD results of the 333-fs-binned data (square), 500-fs-binned data (triangle), and 1-ps-binned data (circle) are plotted for **c** the full and **d** early time domains (<6.5 ps). The first rSVs multiplied by their singular values were fitted with a kinetic function convoluted with an instrument response function with 800-fs FWHM. The fit kinetic function (blue curve) consists of an exponential decay function with a time constant of 8.7 ps (±0.6) and a damped cosine function with a damping time constant of 800 fs (±100) and a wavenumber of 13 cm^−1^ (±1).

Proteins of the globin superfamily can bind and release diatomic molecules such as oxygen and carbon monoxide and serve a critical role in their transportation. Among them, homodimeric hemoglobin (HbI) from the clam *Scapharca inaequivalvis*, which belongs to the globin superfamily, has a unique homodimeric structure. Due to the homodimeric structure, which is advantageous for the study of allostery compared to hemoglobin possessing the heterotetrameric structure, the dynamics of HbI have been intensively investigated (Fig. 1a)^1–8^. Notably, time-resolved experimental techniques such as time-resolved optical spectroscopy^9–12^, time-resolved X-ray crystallography (TRXC)^13–15^, and time-resolved X-ray solution scattering (TRXSS)^16–18^, which is also known as time-resolved X-ray liquidography, have been applied to HbI and its mutants to elucidate the structural transition of HbI upon ligand dissociation. Those studies revealed the structural origin of functional characteristics of HbI, such as cooperativity in ligand binding. Although those studies have clarified the structural dynamics of HbI from the subnanosecond time domain (>~100 ps), ultrafast structural dynamics of HbI still remain elusive. The main reason for such ambiguity is the insufficient time resolutions of the previous studies. In fact, the earliest reaction intermediate (I_1_) identified in the previous TRXSS study with the time resolution of 100 ps has quite a different structure from the initial carboxy structure^16^. However, the detailed transition pathway from the initial carboxy structure to I_1_ remains to be explored. The ultrafast structural dynamics before the formation of I_1_ can give insight not only into the transition to subsequent structural dynamics but also into the unique ultrafast dynamics of HbI. Hence, it is essential to unveil the initial structural response of HbI upon the photodissociation of the ligands.

Femtosecond TRXSS (fs-TRXSS) at X-ray free-electron lasers (XFELs) is one of the promising experimental methods to study the initial structural response of HbI in the solution phase. Indeed, fs-TRXSS has been used to disclose the ultrafast structural dynamics of small molecules^19–25^ and proteins^26,27^. In spite of its strong potential, however, fs-TRXSS has been applied to a limited number of model protein systems such as myoglobin (Mb) and photosynthetic reaction center unlike time-resolved serial femtosecond X-ray crystallography (TR-SFX), which has been used for various protein systems^28–34^. Moreover, the interpretation of fs-TRXSS data is still under debate. For instance, a femtosecond small-angle X-ray scattering (fs-SAXS) study reported the photoinduced underdamped oscillatory change of the radius of gyration (*R*_g_) of Mb^27^. On the contrary, in a subsequent theoretical study, it was argued that such an underdamped oscillatory feature does not originate from the structural change of the protein body but from the hydration shell surrounding the protein since the signal in the SAXS region is influenced by the change of hydration shell as well as the protein itself^35^. Moreover, it was argued that the *R*_g_ change of the bare protein exhibits overdamped behavior rather than underdamped behavior. Although such a critical controversy in the analysis of ultrafast TRXSS data remains, there has been no experimental attempt to settle the argument by directly comparing the analysis results of the SAXS and wide-angle X-ray scattering (WAXS) regions.

In the present work, we studied the initial structural response of HbI upon photoexcitation via fs-TRXSS, covering both SAXS (0.06 ≤ *q* ≤ 0.1 (Å^−1^)) and WAXS (0.13 ≤ *q* ≤ 1.0 (Å^−1^)) regions and compared the global structural changes obtained by analyzing the two regions. The kinetic and structural analyses on the WAXS region of fs-TRXSS data of wild-type HbI(CO)_2_ revealed the existence of an earlier reaction intermediate (I_0_) and the subsequent thermal transition to I_1_ that was reported in the previous TRXSS study^16^. I_0_ exhibits ultrafast coherent motion in addition to the transition to I_1_. The detailed structural changes related to the formation of I_0_, the coherent motion, and the subsequent evolution to I_1_ were clarified via the experiment-restrained rigid-body (ERRB) structure refinement aided by Monte Carlo (MC) simulations^16^. The structural dynamics analysis using the SAXS region of fs-TRXSS data was performed via the Guinier analysis. The global structural change elucidated via analyzing the WAXS region has strikingly different features compared to that revealed by the Guinier analysis. These results indicate that the femtosecond SAXS (fs-SAXS) data does not originate solely from the bare protein, whereas the femtosecond WAXS (fs-WAXS) data is relatively free from the influence of the hydration shell.

Using the fact that the SAXS and WAXS regions contain the information of the hydration shell and bare protein mostly, respectively, we designed an advanced analysis scheme that considers both the changes of the hydration shell and the bare protein. In this analysis scheme, the structural change of the bare protein was refined using the signal in the WAXS region. Then the hydration shell of the protein structures obtained from the WAXS analysis was refined using the signal in the SAXS region. The signal in both the WAXS and SAXS regions were well reproduced when this scheme was used. In addition, by optimizing the electron density of the hydration shell at each time delay, we tracked the change of electron density of the hydration shell over time. The early time profile of the electron density change of the hydration shell was similar to that of the volume change obtained from the Guinier analysis.

To confirm this finding and check whether the observation of the long-lived vibration depending on the *q* range is limited to the HbI case, we revisited the fs-TRXSS data on Mb. The same analysis used for the HbI data was applied to the Mb data, and the results show that the analysis using the WAXS region and the Guinier analysis yielded different features as in the case of HbI, revealing similar dependency on the scattering angle used for the analysis. The fact that two different proteins exhibit similar dependency of the analysis results on the scattering angle used for the analysis indicates that the effect of the hydration shell on fs-SAXS data is a common phenomenon.

## Results and discussion

### Ultrafast kinetics of HbI using the WAXS region

In a previous theoretical study, it was proposed that the signal in the SAXS region can be affected by non-protein factors^35^. Indeed, according to our simulation results, the electron density change of the hydration shell can considerably contribute to the signal in the SAXS region (*q* ≤ 0.1 Å^−1^) (see Supplementary Information and Supplementary Fig. 1 for detailed information). In contrast, its effect on the signal in the wider angle region (*q* ≥ 0.13 Å^−1^) is insignificant. Accordingly, to elucidate the structural change of the protein itself without being significantly compounded by the effect of the hydration shell, we first focused on analyzing the WAXS data by excluding the data with a *q* range <0.13 Å^−1^. The difference in the analysis results due to the *q* range will be discussed later. A caveat is that the specific WAXS ranges used in the current study are not universal. Various factors can affect the overall contribution of the change of the hydration shell to the signal and the range of the WAXS region relatively free from such contribution. For example, the size of protein and the degree of the structural change can influence the relative contribution of the change of the hydration shell to the signal. In addition, the environment surrounding the protein, for example, in the case of a membrane protein, can also affect the relative contribution. Besides, it should be noted that HbI and Mb undergo organized subtle movements of helices, whereas other proteins may undergo large conformational changes, such as partial unfolding or domain movement. In the latter case, the specific WAXS region relatively free from the change of the hydration shell can be different from that for HbI.

**Fig. 2:**
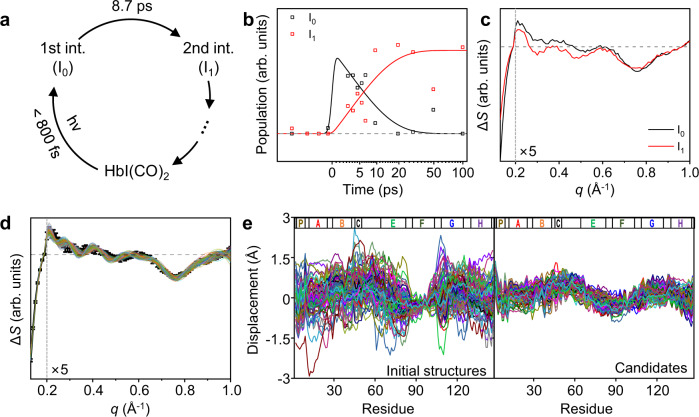
Kinetic analysis and structure refinement for intermediates of HbI. **a** A kinetic model for the initial response of HbI(CO)_2_ upon photoexcitation. The first reaction intermediate (I_0_) forms within the instrument response function (800 fs) and transforms into the second reaction intermediate (I_1_) with a time constant of 8.7 ps. **b** Relative population profiles of I_0_ and I_1_ calculated based on the sequential kinetic model (black and red lines). Optimized populations of each reaction intermediate at each time delay are marked with open squares. **c** SADSs of I_0_ and I_1_. The SADSs were calculated by taking a linear combination of the first and second left singular vectors (lSVs) of SVD results. For clarity, the high *q* region with *q* ≥ 0.2 Å^−1^ is scaled up by 5. **d** The SADS of I_0_ (black) and the theoretical difference scattering curves of 108 candidates (other colors). For clarity, the high *q* region with *q* ≥ 0.2 Å^−1^ is scaled up by 5. **e** Displacement plots of initial structures used for the ERRB structure refinement aided by MC simulations (left) and candidate structures obtained from the structure refinement (right). For each subunit, the displacement was calculated as the changes in the distance from the iron atom to the Cα atom of each residue with respect to the carboxy structure. The displacements of the same residues in the two subunits were averaged. For the displacement plots of the initial structures, 108 initial structures were selected randomly out of the total 270 initial structures used for the structure refinement. The labels of the helices are shown at the top of the plots. The helix labels follow the helix identification in the carboxy structure (PDB entry: 3sdh).

The WAXS regions (0.13 ≤ *q* ≤ 1.0 (Å^−1^)) of the time-resolved difference scattering curves of HbI(CO)_2_ upon photoexcitation are shown in Fig. 1b. To extract the kinetics of structural change from the time-resolved difference scattering curves, the kinetic analysis aided by singular value decomposition (SVD) and the kinetic modeling was applied. In the optimized kinetic model shown in Fig. 2a, the first intermediate forms within our time resolution (~800 fs) and transforms to the second intermediate, with a time constant of 8.7 ps (±0.6) (Fig. 1c). Based on this kinetic model, the population change and species-associated difference scattering curves (SADSs) of the first and second intermediates were obtained (Fig. 2b, c). The SADS of the second intermediate is in good agreement with that of I_1_ obtained from the previous TRXSS study (Supplementary Fig. 4)^16^. Hence, we assigned the second intermediate to I_1_.

The ultrafast dynamics of HbI could not be described solely by the exponential kinetics. The first right singular vector (rSV) exhibits a deviation from the exponential fit in the early time domain (<3 ps). The TRXSS data encrypts the information of both structural change and population kinetics of the protein. The oscillatory feature in addition to the exponential kinetics indicates that a non-thermal motion is involved in the dynamics since the thermally induced dynamics generally exhibit a simple exponential behavior. The SVD on the *q* range from 0.2 to 1.0 Å^−1^ does not exhibit such an oscillatory deviation, and only the small *q* range (0.13 ≤ *q* ≤ 0.2 (Å^−1^)) of the entire WAXS range contributes to this deviation (Supplementary Fig. 5a). Considering the reciprocal relation between the *q* value and the length scale, this result indicates that the oscillatory deviation originates mostly from the overall structural change of the protein rather than localized structural change. The oscillatory deviation can be fitted by a damped cosine function with a damping time constant of 800 fs (±100) and a wavenumber of 13 cm^−1^ (±1) (Fig. 1d). Similar delocalized short-lived vibrational motions with the damping time constants <2 ps have been observed in other biomolecules such as DNA and lysozyme previously using femtosecond optical Kerr effect spectroscopy^36–38^. On the other hand, it was reported that Mb shows long-lived underdamped vibration lasting >5 ps based on the analysis of fs-SAXS data on Mb^35^. The fs-WAXS data of HbI does not show such a long-lived underdamped vibration. As will be discussed later, it turned out that the observation of the long-lived underdamped vibration depends on *q* ranges rather than proteins.

Meanwhile, the overall signal amplitude of the difference scattering curves in the WAXS region rises within the time resolution and thereafter exhibits no considerable amplitude change until 100 ps (Supplementary Fig. 5b). This result indicates that the investigated time window does not exhibit structural change due to the cooperativity or geminate recombination. Under the laser fluence used for our study, ~40% of the population are fully photolyzed in which two heme groups are excited and the others are partially photolyzed in which one of the heme group is excited according to a previous TRXSS study^35^. If the cooperativity would exist, the structural change of one subunit would occur first and subsequently lead to a similar structural change in the other subunit. In that case, since the number of subunits undergoing the structural transition is doubled, the overall amplitude of the difference scattering curve should substantially increase. On the contrary, the overall amplitude of the difference scattering curve should decrease if the geminate recombination occurs, since the number of photolyzed protein molecules decreases. Such considerable amplitude change is not apparent in the TRXSS data.

The kinetic analysis results offer valuable information on the formation and transition of intermediate states and coherent motion. The kinetic information alone, however, cannot visualize the detailed structural change of protein encrypted in the TRXSS data, and thus structural analysis is required.

### Structural dynamics of HbI during the formation of I_0_

To retrieve the structures of the reaction intermediates and obtain structural information on the coherent motion, the ERRB structure refinement aided by MC simulation was used. The structure refinement method was applied to four difference scattering curves (the SADSs of I_0_ and I_1_ and the time-resolved difference scattering curves at 0.5 and 1.5 ps). Since I_0_ exhibits the largest upward and downward deviation from the exponential behavior at 0.5 and 1.5 ps, respectively, I_0_ at 0.5 and 1.5 ps will be labeled as I_0_^U^ and I_0_^D^. The results of the structure refinement for I_0_ are shown in Fig. 2d, e as an example (see Supplementary Information for the refinement results for the other curves). The theoretical difference scattering curves of the candidate structures are in good agreement with the target experimental difference scattering curves (Fig. 2d and Supplementary Fig. 6). In addition, the variation of displacements from the iron atom of the heme group to Cα atoms among the structures decreased after the structure refinement, indicating that the candidates have converged to the optimized structure through the structural refinement (Fig. 2e).

**Fig. 3:**
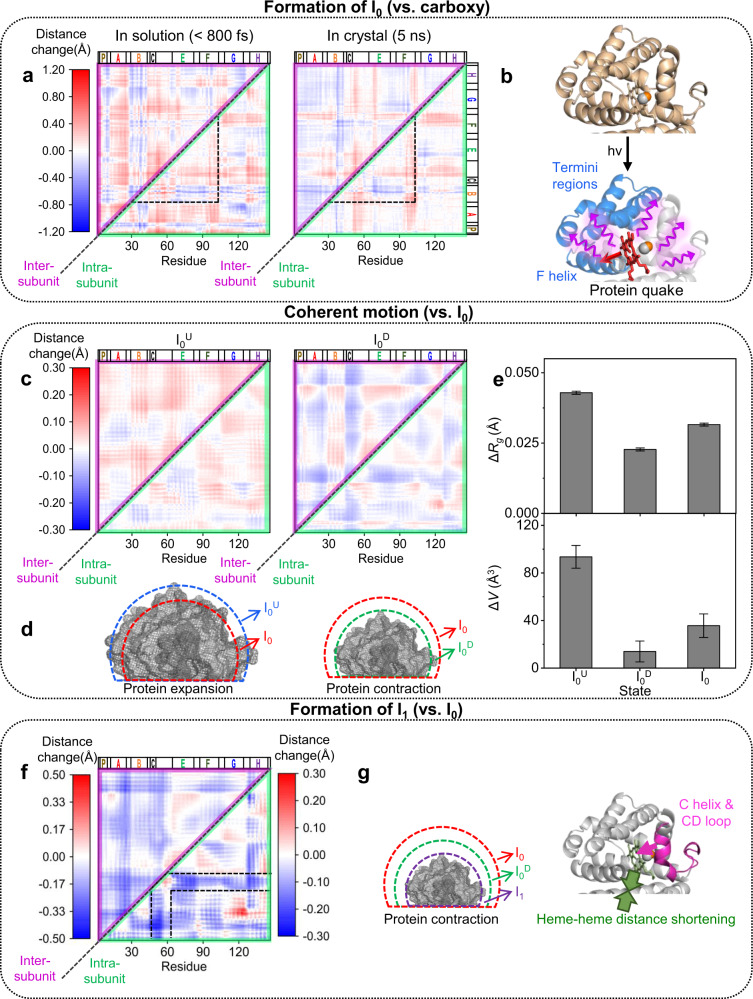
Ultrafast structural dynamics of HbI obtained from the analysis of TRXSS data in the WAXS region. **a** Averaged difference distance maps (DDMs) of I_0_ (left) and the crystal structure at 5 ns after photolysis (right, PDB entry: 2grz). The carboxy structure (PDB entry: 3sdh) was used as the reference structure. The upper triangle and the lower triangle of the DDM are inter-subunit and intra-subunit DDMs, respectively. Two DDMs are shown in the same scale. The region from the B helix to the F helix where the intra-subunit structural changes in the solution phase and the crystalline phase are similar is indicated by dashed lines. **b** A schematic figure for the protein quake. The F helix, the C-terminus, and the N-terminus are indicated with blue color, and the heme group is indicated with red color. The red and purple arrows indicate structural deformation propagation from the heme group to the nearby helices such as the F helix and from the nearby helices to the entire protein including the termini regions, respectively. **c** Averaged DDMs of I_0_^U^ and I_0_^D^. I_0_ was used as the reference. Two DDMs are shown in the same scale. **d** Schematics for the expansion and contraction of HbI. The volumes of I_0_, I_0_^U^, and I_0_^D^ are indicated with red, blue, and green dashed lines, respectively. The volumes of HbI after volume change are indicated by gray meshes. **e** The difference of radius of gyrations (Δ*R*_g_) and volumes (Δ*V*) of the candidate structures of I_0_^U^, I_0_^D^, and I_0_. All differences were calculated with respect to the carboxy structure. Data is presented as mean values ± SEM. The number of candidate structures used for the analysis were 108, 95, and 94 for I_0_, I_0_^U^, and I_0_^D^, respectively. **f** Averaged DDM of I_1_. I_0_ was used as the reference. The color gradient for the inter-subunit DDM is shown on the left and that for the intra-subunit DDM is shown on the right. The C helix and CD loop regions that show large movement are indicated by dashed lines. **g** Schematics for the structural change of HbI during the transition from I_0_ to I_1_. The left panel shows the contraction of the protein. The volumes of I_0_, I_0_^D^, and I_1_ are indicated with red, green, and purple dashed lines, respectively. The volume of HbI after volume change is indicated by gray mesh. The right panel shows the movement of the C helix and the CD loop toward the center of the protein (magenta arrow) and the contraction of the heme–heme distance (green arrows) during the transition to I_1_. In **d**, **g**, exaggerated volume changes are shown for clarity.

To inspect the tertiary and quaternary structures of the candidate structures simultaneously, we utilized difference distance maps (DDMs), which display the residue–residue distance differences between two protein structures. The intra- and inter-subunit DDMs can represent the tertiary and quaternary structural changes, respectively (see Supplementary Information for details on the calculation of a DDM). For the DDMs of reaction intermediates (I_0_, I_0_^U^, I_0_^D^, and I_1_), the averaged DDMs obtained by averaging the DDMs of the candidate structures of reaction intermediates were used for the analysis. To compare the structural change of HbI in solution and crystalline phase during the photoreaction, the DDM of I_0_ with respect to the carboxy structure is compared with that of the crystal structure of HbI at 5 ns after photolysis as shown in Fig. 3a^13^. In the previous TRXSS study, similar structural changes of HbI in the solution and crystalline phases were identified in the late time domain (>100 ns)^16^. Our fs-TRXSS data reveals that the intra-subunit helix movement similar to that in the crystalline phase at a much later time delay (5 ns) was already developed even within a picosecond. For example, the Pearson correlation coefficient between the intra-subunit DDMs of I_0_ and the crystal structure at 5 ns using the region from the B helix to the F helix is 0.77, which falls into a range typically indicating a strong correlation between two variables (see Supplementary Information for detailed information on the calculation of Pearson correlation coefficient). Similar intra-subunit structural changes in I_0_ and the crystalline phase indicate that the helix movements such as the clamshell motion of the E and F helices observed in crystallographic studies also occur during the formation of I_0_ in the solution phase^15^. However, the regions excluding the region from the B helix to the F helix show a small Pearson correlation coefficient of 0.24, which indicates that N- and C-terminus regions show different movement in the solution and crystalline phase. The overall intra-subunit distance differences of the residues nearby N- and C-terminus regions are larger in I_0_ than in the crystal. This feature can be rationalized by the fact that the movements of such residues are less constrained in solution. The inter-subunit DDMs of I_0_ and the photolyzed crystal structure exhibit different features as well. Positive inter-subunit difference distances are observed more frequently in I_0_ than in the photolyzed crystal structure. This result indicates that the protein size compared to the carboxy structure increases more in I_0_ than in the crystalline phase. Besides, the heme–heme distance is longer in I_0_ than in the photolyzed crystal structure (Supplementary Fig. 7a).

It is worth noting that the structural change propagates rapidly within a picosecond into the termini regions located far from the heme group where the reaction is initiated (Fig. 3a, left panel). Moreover, we found that the overall structural change of I_0_ was also accompanied within this ultrafast time range, as can be seen from the increase of the *R*_g_ and protein volume (see Supplementary Information for detailed information on the calculation of *R*_g_ and volume and Supplementary Fig. 7b, c). We propose that such rapid and global structural changes of HbI can be rationalized by the protein quake (Fig. 3b). According to the protein quake hypothesis, structural deformation as well as excessive energy emerges at a focus (epicenter), and subsequently, the structural deformation spreads into the entire protein in a quake-like manner inducing a global structural change^39–43^. If we introduce this concept to HbI, the focus of the protein quake is the heme group, and the nearby helices such as the F helix located in the vicinity of the heme group would be disturbed most quickly upon ligand dissociation^44^. The rearrangements of such helices can deliver the structural deformation of the focus to the entire protein through the protein quake leading to an ultrafast global structural change. We note that the protein quake hypothesis has been introduced for several proteins to describe ultrafast global structural changes^26,27^, but the process of the protein quake has been rarely explained by tracking specific structural changes in the solution phase. In this work, we visualized detailed helix rearrangements related to the protein quake process, such as the movements of the termini regions and the clamshell motion of the E and F helices.

Meanwhile, the intra-subunit helix movement of HbI during the formation of I_0_ is similar to the helix movement of Mb at 0.5 ps revealed by the TR-SFX study (Supplementary Fig. 8b). Even though the analogous structural changes of HbI and Mb in subnanosecond time regime (~100 ps) after ligand dissociation were observed previously^15^, the ultrafast structural changes that occur within 100 ps have not been compared so far. Similar helix movements of HbI and Mb in the ultrafast time domain indicate a common initial structural response of HbI and Mb upon ligand dissociation. Considering the analogous structures and identical cofactor (the heme group) of HbI and Mb, their comparable initial responses are not surprising.

### Coherent motion of HbI in the early time domain (<3 ps)

As marked by the oscillatory deviation from the exponential decay observed in the kinetic analysis, I_0_ undergoes coherent motion in the early time domain (<3 ps). To reveal the detailed tertiary and quaternary structural changes related to the coherent motion, we performed the structure refinement for I_0_^U^ and I_0_^D^. The structures of I_0_^U^ and I_0_^D^, where the deviations are the largest, can represent the transient structural changes during the coherent motion. Both intra- and inter-subunit DDMs with respect to I_0_ indicate that the majority of residue–residue distances are elongated in I_0_^U^ compared to I_0_, whereas they are shortened in I_0_^D^ (Fig. 3c). These results indicate that the expansion and contraction of the protein occur in I_0_^U^ and I_0_^D^, respectively (Fig. 3d). Considering the expansion and contraction of the protein during the coherent motion, the coherent motion can be identified as a breathing motion of HbI^45–48^.

To quantify the expansion and contraction identified by the helical motion in more detail, we scrutinized the change of the overall protein size during the coherent motion by calculating *R*_g_ and protein volume of I_0_^U^ and I_0_^D^. The *R*_g_ and volume of I_0_^U^ and I_0_^D^ are larger and smaller than those of the I_0_, respectively (Fig. 3e). Such changes of the *R*_g_ and volume are well correlated with the changes of the residue–residue distances during the coherent motion shown in Fig. 3c. The trends of the *R*_g_ and volume change are also reproduced in the amplitude change of the difference scattering curve at the smallest *q* (*q* = 0.13 Å^−1^) used for the WAXS analysis (Supplementary Fig. 9). The similar trends between the change of the protein size and the amplitude at the smallest *q* are in accordance with the fact that the signal in the small *q* region reflects the global structural change of proteins.

### Structural dynamics of HbI during the transition from I_0_ to I_1_

The DDMs between I_1_ and I_0_ are shown in Fig. 3f. The residues in the C helix and CD loop exhibit dominant intra-subunit movement during the transition from I_0_ to I_1_. The movements of the C helix and CD loop region can be induced by the rearrangement of the E helix during the formation of I_0_ since the E helix is connected to the CD loop region. The flexibility of the CD loop region may be responsible for the large movement. The negative intra-subunit distance changes of the C helix and CD loop indicate that they moved toward the center of the protein. The movement of the C helix and CD loop toward the center of the protein can also be confirmed by the displacement plot between I_1_ and I_0_ (Supplementary Fig. 10). A similar displacement of the CD loop has been reported in the TRXC study on HbI^13^. Since the intra-subunit movement in the termini regions is not significant during the transition from I_0_ to I_1_, the movement of termini regions with respect to the carboxy structure is still larger in I_1_ than in the crystalline phase at 5 ns after photolysis, as in the case of I_0_ (Supplementary Fig. 11). In terms of the quaternary structure, overall distance contraction in DDMs implies that the protein size decreases during the transition. Indeed, the *R*_g_ and volume of the protein decrease during the transformation of I_0_ to I_1_ (Supplementary Fig. 7b, c). The distance between the heme groups also decreases further during the transformation from I_0_ to I_1_ (Supplementary Fig. 7a). A schematic for the structural change during the transition from I_0_ to I_1_ is shown in Fig. 3g.

In contrast to the similar structural change observed in I_0_ of HbI and the crystal structure of Mb at 0.5 ps including that in the region between the C helix and D helix (Supplementary Fig. 8b), the structural change of Mb in later time domain (>5 ps) shows differences to that of HbI. According to the TR-SFX study on Mb, the movement of the E helix extends up to 150 ps, whereas the movement of the C and D helices is relatively not significant. In contrast, in HbI, the movement of the C helix and CD loop region is apparent up to 100 ps with the formation of I_1_, whereas the movement of the E helix is not large. We note that the D helix of HbI is not well structured and exists as a part of the CD loop. The difference in the structural change observed between Mb by TR-SFX and HbI by fs-TRXSS may arise from the crystal packing and the different rigidity of the C and D helices in HbI and Mb.

### Comparison of the structural dynamics of HbI and Mb in the solution phase

For comparison with HbI, we analyzed the WAXS region of the previously reported TRXSS data on Mb using the same approach used for HbI (see Supplementary Information and Supplementary Figs. 13 and 14 for detailed information). As expected from the structural similarity between Mb and HbI, the structure refinement results indicate that Mb exhibits similar global structural change to HbI. For example, as in the case of HbI, the *R*_g_ and volume of Mb increase during the formation of the first reaction intermediate, indicating the expansion of the protein (Supplementary Fig. 14d, e). The ultrafast and global structural change of Mb implies that the initial structural response of Mb is a consequence of the protein quake, as in the case of HbI. During the transition from the first to the second intermediate, the overall protein size decreases as indicated by the decrease of *R*_g_ and protein volume (Supplementary Fig. 14d, e). Such a trend of global structural change, the rapid expansion and then subsequent contraction of overall protein size, is similar to that observed in HbI.

### Comparison of analysis results using WAXS and SAXS regions

**Fig. 4:**
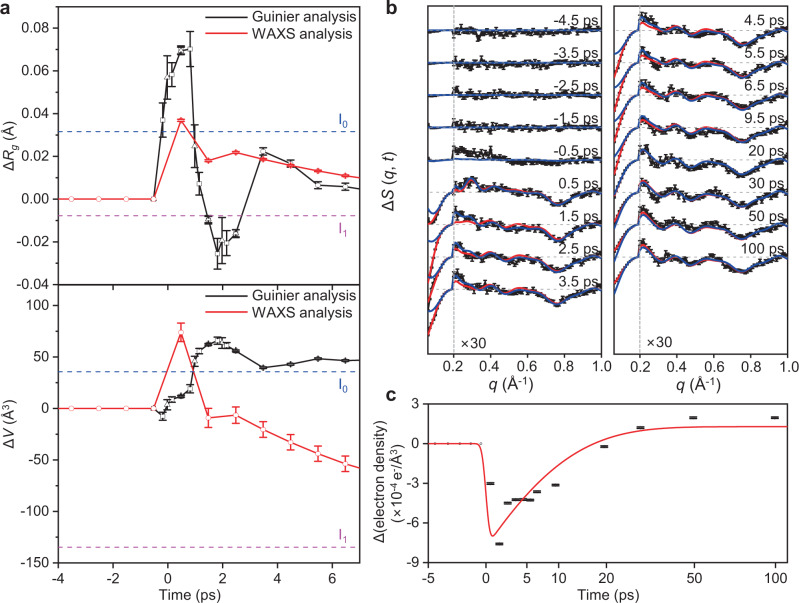
Analysis of TRXSS data of HbI in the SAXS region using the Guinier analysis and the temporal change of the electron density in the hydration shell. **a** Time profiles of the radius of gyration change (Δ*R*_g_) and volume change (Δ*V*) with respect to the carboxy structure in the early time domain (<7 ps) after photoexcitation obtained from the Guinier analysis on the SAXS region (black) and WAXS analysis (red). For the Guinier analysis results, the results from the 333-fs-binned data, 500-fs-binned data, and 1-ps-binned data are plotted with square, triangle, and circle, respectively. The *R*_g_s and volumes of I_0_ and I_1_ are marked with blue and magenta dashed lines, respectively. For the Guinier analysis results, the error bars represent the fitting errors. For the WAXS analysis results, error bars represent SEM. **b** The experimental femtosecond TRXSS data and its SEM (black), the calculated difference scattering curves after fitting the electron density of the hydration shell (red), and the calculated difference scattering curves before the fitting (blue) from −4.5 to 100 ps. For clarity, the high *q* region with *q* ≥ 0.2 Å^−1^ is scaled up by 30. **c** The change of electron density of the hydration shell as a function of time (black) and an exponential decay function with a time constant of 8.7 ps (red).

To compare the structural changes extracted from the SAXS and WAXS data, the time profiles of Δ*R*_g_ and Δ*V*, the changes of *R*_g_ and volume with respect to the carboxy structure, are plotted in Fig. 4a. The time profiles of ∆*R*_g_ and ∆*V* for the WAXS region were obtained using the linear combination of ∆*R*_g_s and ∆*V*s of the reaction intermediates calculated using their candidate structures from the structure refinement using the WAXS region (see Supplementary Information for detailed information on the time profile calculation using fs-WAXS data). The time profiles of ∆*R*_g_ and ∆*V* for the SAXS region were obtained using the Guinier analysis on 0.06 ≤ *q* ≤ 0.1 Å^−1^ (Supplementary Fig. 15). The time profiles of Δ*R*_g_ and Δ*V* in the early time domain (<7 ps) obtained by analyzing the SAXS and WAXS regions exhibit apparently different features. The time profile of Δ*R*_g_ of the SAXS analysis over time (Δ*R*_g_^SAXS^) has an oscillatory feature with a much larger amplitude and damping time than those of the WAXS analysis (Δ*R*_g_^WAXS^). Also, Δ*R*_g_^SAXS^ oscillates around zero, whereas Δ*R*_g_^WAXS^ is positive. Our analysis results indicate that the time profiles of Δ*R*_g_^SAXS^ and Δ*R*_g_^WAXS^ resemble the underdamped and overdamped behaviors, respectively, as proposed in the theoretical study^35^. Likewise, two time profiles of Δ*V* obtained from the SAXS and WAXS analyses, Δ*V*^SAXS^ and Δ*V*^WAXS^, respectively, exhibit strikingly different behaviors. Δ*V*^SAXS^ is always positive after time zero. In contrast, Δ*V*^WAXS^ becomes negative after 1.5 ps since the volume of I_1_ is ~150 Å^3^ smaller than the carboxy structure. The difference of Δ*V*^SAXS^ and Δ*V*^WAXS^ in the late time delays may indicate that the electron density of the hydration shell does not relax into that of the carboxy structure yet, since Δ*V*^SAXS^ and Δ*V*^WAXS^ would be the same if the electron density of the hydration shell fully relaxes into that of the carboxy structure. In this regard, it is expected that Δ*V*^WAXS^, which is less affected by the change of the hydration shell, would be appropriate for representing the volume change of the bare protein in the late time delays. Besides, the rises of Δ*R*_g_^SAXS^ and Δ*V*^SAXS^ exhibit different features compared to the rise of Δ*R*_g_^WAXS^ and Δ*V*^WAXS^. The rise of Δ*V*^SAXS^ after photoexcitation is delayed compared to that of Δ*R*_g_^SAXS^, whereas the rises of Δ*V*^WAXS^ and Δ*R*_g_^WAXS^ are almost concurrent in our time resolution (~800 fs). One possible explanation for such a delayed rise of ∆*V* compared to ∆*R*_g_ can be the anisotropic change of the protein and hydration shell. In a previous theoretical study on Mb, it was suggested that the protein quake propagates anisotropically rather than isotropically^35^. Such anisotropic change of the protein and hydration shell can induce different temporal behavior of ∆*R*_g_ and ∆*V*.

The delay of the Δ*V*^SAXS^ rise compared to that of Δ*R*_g_^SAXS^ was also observed in the previous TRXSS study on Mb using the SAXS region^27^. In the previous theoretical study, it was argued that such a phenomenon occurred when both the structural change of the bare protein and the hydration shell were included in the simulation^35^. These observations on the difference in the rise of Δ*V* and Δ*R*_g_ obtained from the SAXS and WAXS regions demonstrate that the signal in the SAXS region contains the contribution from the hydration shell as well as the bare protein.

Based on the fact that the WAXS and SAXS regions are dominantly affected by the change of the bare protein and non-protein factors such as the hydration shell, respectively, we tracked the change of the electron density of the hydration shell by fitting the signal in the SAXS region (see Supplementary Information for detailed information of the SAXS fit). Since the signal in the WAXS region is sensitive to the structural change of the bare protein mostly, during the fitting, the structures of the reaction intermediates were fixed to the candidate structures obtained from the WAXS region analysis. Then the signal in the SAXS region was fitted by refining the electron density of the hydration shell used for the theoretical difference scattering curve calculation of the candidate structures. As shown in Fig. 4b, the agreement between the calculated and experimental difference scattering curves in the SAXS region is enormously improved by refining the electron density of the hydration shell without impairing the agreement in the WAXS region. These results demonstrate that the signal in the SAXS region is dominantly affected by the change of the hydration shell, whereas the signal in the WAXS region is affected mostly by the structural change of the bare protein. In addition, the change of electron density of the hydration shell over time was experimentally captured by refining the electron density of the hydration shell at each time delay. Upon photoexcitation, the electron density contrast of the hydration shell compared to the bulk solvent decreases up to ~2.5%, Such a change is equivalent to ~0.2% decrease of the electron density of the hydration shell. Then the change of the electron density follows the formation of I_1_, as it can be described with an exponential decay function with its time constant fixed at 8.7 ps obtained from the WAXS analysis (Fig. 4c).

In contrast to the negative difference of the hydration shell density in the early time delay observed here, a positive difference of the hydration shell density upon photoexcitation depending on the distance from the protein surface was proposed in a theoretical study on Mb^35^, which tracked the time-dependent change of the electron density of the hydration shell ranging to the region apart from the protein >1 nm. Since CRYSOL, the program used to calculate the scattering curve, describes the hydration shell as a layer with a thickness of 3 Å^49^, the density change dependent on the distance from the protein surface is approximated as a constant density of a single hydration shell instead of the density changes of multiple shell layers. To check the effect of this approximation, we performed a series of simulations considering the effect of multiple shell layers and their density changes (see “Single-layer model vs. multiple-layer model to describe the density change in the hydration shell” section of Supplementary Information for detailed information). The simulations confirm that the difference scattering curve due to the density changes of multiple shell layers can be approximated with the difference scattering curve due to the density change of a single shell layer, confirming that this approximation should still capture the general density change (Supplementary Fig. 12a, b). The simulations also show that the approximated density change of the single shell layer is weighted toward those of closer layers, confirming that the density in the closer vicinity to the protein body has a higher contribution to the difference scattering curve (Supplementary Fig. 12c, d). If the simulated difference scattering curves using the time-dependent density change profiles proposed in the theoretical study are approximated with difference scattering curves using a single shell layer, the approximated density changes show positive density changes at most time delays including early ones. If the propagation of the positive density shock similar to that proposed in the theoretical study indeed occurs, the lack of the transient positive density change in our data may be because the positive shock moves away from the protein too fast to be captured with the time resolution of the current study. Otherwise, our result does not quantitatively agree with the density change profiles proposed in the theoretical study.

The dominant effects of the hydration shell and bare protein to the SAXS and WAXS region, respectively, were confirmed by the time profiles of multiple structural parameters. For example, Δ*V*^SAXS^ and the change of the electron density of the hydration shell exhibit a similar rising time (Supplementary Fig. 16a), indicating that the delayed rise of Δ*V*^SAXS^ may originate from the change of hydration shell and the SAXS region is dominantly affected by the change of the hydration shell. In contrast, the time profile of Δ*R*_g_^WAXS^ resembles the first rSV obtained from the SVD analysis on the WAXS region, which implies that the structural change of bare protein largely influences the WAXS region (Supplementary Fig. 16b).

To confirm the generality of the effects of the hydration shell on the SAXS region, we analyzed the WAXS region of fs-TRXSS data of Mb and compared the results with the results of the Guinier analysis. In a previous fs-TRXSS study on Mb using the SAXS region, a long-lived underdamped vibration was identified by the Guinier analysis. We analyzed the WAXS region of the same data of Mb (0.17 ≤ *q* ≤ 1.0 (Å^−1^)). In contrast to the Guinier analysis results, the rSVs obtained from the analysis of the WAXS region do not exhibit oscillatory features (Supplementary Fig. 13), indicating that the long-lived underdamped vibration is absent when the WAXS region is analyzed. The different analysis results obtained from the WAXS and SAXS regions of the Mb data show good agreement with the results obtained from the analysis on HbI. The fact that two different proteins exhibit similar dynamics dependence supports that the effect of non-protein factors on the SAXS region of fs-SAXS data is a general phenomenon. Meanwhile, unlike the case of HbI, the rSVs of the WAXS region of the Mb data do not indicate the emergence of a coherent motion manifested as an oscillatory deviation in the early time domain. Considering the existence of the coherent motion of HbI, which is a similar heme protein, it seems that the coherent motion of Mb dampens within the time resolution rather than it does not undergo any coherent motion^50^. For example, the TR-SFX study on Mb reported the coherent motion corresponding to some inter-residue distances^28^. A possible explanation for the ultrafast damping can be the larger ratio between surface area and volume in Mb. The surface area relative to the volume is larger in Mb since Mb and HbI have monomeric and dimeric structures, respectively. The larger relative surface area may lead to faster damping of a coherent motion via larger interaction with the hydration shell so that the coherent motion was not detected with the experimental time resolution.

The dependence of the temporal behavior on the SAXS and WAXS regions implies that the fs-SAXS data is dominantly affected by non-protein factors, such as the hydration shell. Here we experimentally demonstrated that ultrafast time-resolved SAXS data encrypts the additional factors as well as the structural change of the protein molecule itself by comparing the structural dynamics decoded from the SAXS and WAXS regions of HbI and Mb. Furthermore, we tracked the change of the hydration shell and protein structure over time using an analysis method that refines the latter using the WAXS region and the former using the SAXS region.

**Fig. 5:**
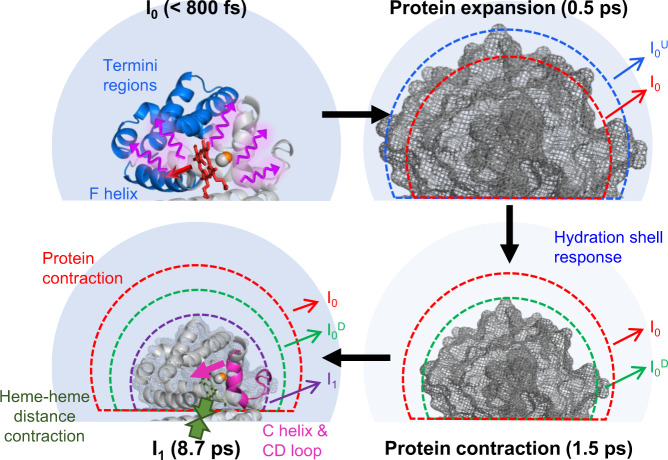
Schematic for the initial response of HbI(CO)_2_ upon photoexcitation. (Left upper panel) The photodissociation of carbon monoxide induces the structural change of the nearby helices such as the F helix that binds the heme group. The structural deformation in these helices is transmitted to C- and N-terminus regions within ~800 fs. (Right upper and right lower panels) In the early time domain, the protein undergoes a non-thermal coherent motion, which leads to the expansion and contraction of the protein similar to a breathing motion. The electron density of the hydration shell, which is indicated by the clarity of the color in the light blue circle around the protein, decreases up to ~1.5 ps after photoexcitation. (Left lower panel) Then the thermal motion of helices leads to the transformation of I_0_ to I_1_, accompanying the movement of the C helix and CD loop toward the heme group, the contraction of volume, the shortening of the heme–heme distance, and the change of the electron density of the hydration shell.

The initial response of HbI upon ligand dissociation revealed by the analysis of both the WAXS and SAXS regions is summarized in Fig. 5. The earliest reaction intermediate identified in this study, I_0_, is formed within our time resolution (~800 fs) and evolves to I_1_ with a time constant of 8.7 ps. The oscillatory deviation from the exponential kinetics appeared during the early time domain (<3 ps). The oscillatory deviation arises from the coherent motion of I_0_ involving the overall expansion and contraction. During the formation of I_0_, intra-subunit structural change of helices such as the E and F helices similar to that observed in the crystalline phase at 5 ns occurs. However, unlike the helix movement in the crystal, the large and ultrafast movements of both termini regions were identified. The ultrafast global structural change during the formation of I_0_, which is characterized by ultrafast protein size change as well, implies that the ultrafast structural dynamics of HbI are driven by the protein quake. During the transition from I_0_ to I_1_, the movement of the C helix and CD loop to the center of the protein was observed. Besides, the contraction of the protein size and heme–heme distance occurs during the formation of I_1_. In terms of the hydration shell, the electron density contrast of the hydration shell compared to the bulk solvent initially decreases by ~2.5% up to ~1.5 ps after photoexcitation. Such a decrease is equivalent to ~0.2% in terms of the electron density of the hydration shell. Then it changes along with the formation of I_1_.

In summary, by analyzing both WAXS and SAXS regions of fs-TRXSS data, we studied the ultrafast structural dynamics of HbI(CO)_2_ in the solution phase. For the WAXS region analysis, kinetic analysis aided by SVD and kinetic modeling was used to elucidate the ultrafast kinetics of HbI(CO)_2_ upon photoexcitation. The structures of reaction intermediates and the structural change during the coherent motion were captured via the ERRB structure refinement aided by MC simulations. Consequently, we revealed the structural dynamics of HbI in the solution phase in the ultrafast time domain. Compared to the structural change in the crystalline phase, I_0_ exhibits distinct structural characteristics such as the large movement termini regions, implying the effect of the crystal packing on the structural changes in the crystalline phase. Such an ultrafast structural change of I_0_ spread over the entire protein indicates that we captured the protein quake process. These results highlight the importance of investigating the structural dynamics of proteins in the solution phase, which is similar to the physiological conditions. Furthermore, with the aid of an excellent spatiotemporal resolution of fs-TRXSS, the coherent motion of HbI was observed, and detailed structural change during the coherent motion was visualized successfully.

The structural analysis results of the SAXS regions using the Guinier analysis were considerably different from those obtained from the WAXS region analysis. These results suggest that one should take into account that the observed dynamics retrieved from fs-SAXS contains not only the changes of bare macromolecules but also those of hydration shells. Based on this argument, by fitting the SAXS region, we tracked the hydration shell change of the protein structures obtained from WAXS region analysis. Furthermore, the fitting result visualizes the delayed change of the electron density of the hydration shell compared to the structural change of the bare protein, which was observed in comparison between the *R*_g_ and volume changes obtained from the Guinier analysis and the WAXS region analysis. These results demonstrate the effects of the hydration shell change on the signal in the SAXS region.

In addition to HbI, we analyzed the structural dynamics of Mb, which can offer invaluable information on the effect of oligomeric state on the structural dynamics. The ultrafast structural dynamics of Mb exhibit both similar and different features to that of HbI. The change of overall protein size upon excitation was similar as it can be deduced from the similar tertiary structures and the same cofactor of Mb and HbI. However, the coherent motion was not observed in Mb. This result indicates that the interaction between the protein and hydration shell can be affected by the oligomeric state since the formation of the oligomer can alter the fraction of protein surface exposed to the solvent.

## Methods

### Data acquisition

The fs-TRXSS data for wild-type HbI(CO)_2_ were collected at the X-ray Pump-Probe endstation of Linac Coherent Light Source using a pump–probe scheme. A flow cell system was exploited for the measurement. In the flow cell system, the HbI(CO)_2_ sample with a concentration of ~0.6 mM was flowed through a capillary back and forth using a syringe pump. The whole flow cell system was saturated with carbon monoxide to maintain the concentration of the ligated form of HbI. At the center of the capillary, the sample was excited using a circularly polarized femtosecond laser pulse with a 532-nm center wavelength. At the sample position, the laser was focused on 200 × 200 μm^2^, and the fluence of the laser was ~0.5 mJ/mm^2^. A femtosecond X-ray pulse with 9.5 keV photon energy and 100 × 100 μm^2^ size at the sample position followed the laser pulse to investigate the structural dynamics of HbI(CO)_2_ after photoexcitation. The scattered X-ray was collected using an area detector (CSPAD). The scattering images were collected at the following time delays: −20, −4.5, −3.5, −2.5, −1.5, −0.5, 0.5, 1.5, 2.5, 3.5, 4.5, 5.5, 6.5, 9.5, 20, 30, 40, 50, 100 ps. A previous study reported that the difference scattering signal is practically isotropic when the sample is excited by a circularly polarized laser pulse^51^. Hence, a scattering image was azimuthally integrated as a function of the momentum transfer vector (*q* = 4*π*sin(2*θ*/2)/*λ*, where 2*θ* is the scattering angle and *λ* is the wavelength of the X-ray) to yield a scattering curve. To extract the information of time-dependent structural change, time-resolved difference scattering curves were generated by subtracting a scattering curve collected at −20 ps from those collected at other time delays. For each time delay, ~20,000 difference scattering curves were averaged to yield an averaged difference scattering curve to achieve sufficient signal-to-noise ratio (SNR) for the data analysis. For fs-WAXS data analysis, the region with *q* > 1.0 Å^−1^ and the SAXS region with *q* < 0.13 Å^−1^ were excluded for further analysis because the former is affected significantly by the structural change of the bulk solvent due to the solvent heating and the latter can be influenced by the change of the hydration shell (Supplementary Fig. 1)^35^. For the fs-SAXS data analysis, the SAXS region with 0.06 ≤ *q* ≤ 0.1 Å^−1^ was used for the analysis.

### Kinetic analysis of fs-WAXS data

First, time-resolved difference scattering curves were subjected to SVD to determine the number of reaction intermediates and their kinetics. SVD can decompose time-resolved experimental data into three components, which are left singular vectors (lSVs), right singular vectors (rSVs), and the singular values. For TRXSS data, lSVs are the time-independent difference scattering curves and rSVs are the time profiles of the lSVs. Each singular value indicates the relative contribution of each singular vector to the experimental signal. From the SVD results, two SVD components were classified as the major components as they predominantly contribute to the experimental signal (Supplementary Fig. 2a–c). According to the number of the major SVD components, two reaction intermediates were identified. The time constant for the transition between these two intermediates was obtained by fitting the reconstructed experimental data of the SVD results to the original experimental data. The data were reconstructed as follows. Each of the first and second rSVs was simultaneously reconstructed using an exponential decay function convoluted with an instrument response function (IRF) represented by a Gaussian function (Supplementary Fig. 3a, b). To consider the contributions of the rSVs to the experimental data, the rSVs scaled by their singular values were reconstructed instead of the rSVs themselves. The reconstructed experimental data were obtained by multiplying the matrix whose columns are the lSVs (**U**) and the matrix whose columns are the reconstructed rSVs (**SV**_reconst_) as follows.

1 $$\varDelta {S}_{{\rm{reconst}}}={\mathbf{{U}}}\times {\mathbf{{SV}}}_{{\rm{reconst}}}$$

We note that the first two lSVs were used for the data reconstruction in this case since two reaction intermediates were identified. The fitting was performed so that the discrepancy between the original data and reconstructed data (Eq. (2)) was minimized.

2 $${\chi}^{2}=\frac{1}{N-p-1}\mathop{\sum}\nolimits _{t}\mathop{\sum}\nolimits _{q}{\left(\frac{\Delta {S}_{\exp }(q,t)-\Delta {S}_{{\rm{reconst}}}(q,t)}{\sigma (q,t)}\right)}^{2}$$

where *N* is the total number of data points used for the fitting and *p* is the number of fitting parameters.

Since the first two rSVs exhibit large deviations from the exponential behavior at 0.5 and 1.5 ps, those two time points were excluded from the exponential decay fit. The *χ*^2^ values are 1.83 when the data collected at 0.5 and 1.5 ps were excluded and 2.47 when those data were included for the fitting.

Then kinetic modeling was performed based on the results of the SVD analysis. In the kinetic modeling, the data are reconstructed using the SADSs and the concentration profiles of the reaction intermediates. The SADS are obtained by taking a linear combination of lSVs, and the concentration profiles are calculated based on a given kinetic model. During the fitting, the coefficients of the linear combination are optimized so that the discrepancy between the experimental data and the reconstructed data is minimized in a similar way used for the analysis of the SVD results. Using the time constants obtained from the analysis of the SVD results, a sequential kinetic model was established. In the sequential kinetic model, the first reaction intermediate, which emerges within our time resolution, transforms to the second reaction intermediate with a time constant of 8.7 ps. The SADSs of the two reaction intermediates were obtained by generating a linear combination of the first and second lSVs based on the sequential kinetic model (Supplementary Fig. 3c). More details for kinetic analysis aided by SVD and kinetic modeling were reported previously^16^.

Even though we observed clear deviations at 0.5 and 1.5 ps from the exponential behavior, the kinetic parameters related to the deviations could not be determined accurately due to the limited number of time points during the deviation. To increase the number of the time points during the deviation and determine the kinetic parameters related to the deviation more accurately, we re-binned the original fs-WAXS data from −0.5 to 2.5 ps with 500-fs time bins and 333-fs time bins. For the kinetic analysis, the first rSVs of the two re-binned datasets were simultaneously fitted with a sum of an exponential function and a damped cosine function convoluted with the IRF of 800-fs full width half maximum (FWHM), as shown in Fig. 1c, d. We note that only the first rSVs were used for the kinetic fit due to insufficient SNR of the second rSVs of 500-fs-binned data and 333-fs-binned data, as shown in Supplementary Fig. 2. During the fitting, all parameters related to the exponential decay were fixed to the values obtained from the prior exponential decay fit of the original 1-ps binned data. Similar to the case of prior SVD fitting, the SVD fitting was performed so that the sum of the differences between the original data and reconstructed data of two re-binned datasets was minimized. All the fittings and error analyses were performed using the MINUIT software packages provided by CERN^52^. We note that, since the original 1-ps binned data have better SNR, they were used for the structure refinement to improve the quality of the structural analysis.

### The ERRB structure refinement aided by MC simulations on fs-WAXS data

In the ERRB structure refinement aided by MC simulations, the template structure of the photolyzed HbI(CO)_2_ was divided into 18 rigid bodies according to their 8 helices and 1 heme group in each subunit of HbI. The TR-SFX study on Mb, a representative heme protein of a monomeric form, revealed that the doming of the heme group and the partial displacement of the iron atom after photolysis were observed within ~250 fs^28^. Considering the time resolution of our experiment (~800 fs), it is reasonable to assume that the heme doming should have been completed even in the first reaction intermediate (I_0_) identified in this study. Hence, the crystal structure of HbI(CO)_2_ at 5 ns after photolysis (PDB entry: 2grz), which exhibits the heme doming, was used as the template structure. The initial structures for the structure refinement were generated by randomly moving the rigid bodies of the template structure via MC simulation method. For the structure refinement of the second reaction intermediate (I_1_), the number of water molecules in the interface of the subunits was fixed to nine following the previous assignment^16^. Otherwise, it was fixed to 11, which is the number of water molecules in HbI(CO)_2_. Even though the structure of I_1_ was previously reported, here we refined the structure again using the TRXSS data obtained in this experiment, expecting that the TRXSS data obtained at the XFEL would give more accurate and precise structural information for the following reasons. First, the SNR of the TRXSS data obtained in this study is higher than that of previous TRXSS data. Second, the X-ray energy bandwidth, which can blur the structural information when it is polychromatic, was more polychromatic in the previous study^16^. The X-ray energy bandwidth of the X-ray used for the previous TRXSS study was ~4% FWHM, whereas the X-ray used for the current study is pseudo-monochromatic.

The initial structures were refined to minimize the target function by moving the rigid bodies of the initial structures. X-ray scattering curves were calculated using CRYSOL version 2.6 after each movement to generate the theoretical difference scattering curves, which are the differences between the scattering intensities of the protein structures under refinement and the carboxy structure (PDB entry: 3sdh)^49^. Previously, it was demonstrated that CRYSOL could be used to calculate the X-ray scattering curves at a wide angle of up to 1 Å^–1^ by employing a large number of harmonics^16^. The target function was composed of the *χ*^2^ term, which describes the disagreement between the theoretical and experimental difference scattering curves, a collision-avoiding term that implies physical plausibility, and a subunit-symmetry constraint term. The forces applied to the rigid bodies were calculated by taking the first derivative of the target function with respect to the position of rigid bodies. Each rigid body was translated and rotated based on the force applied to that rigid body so that the target function was allowed to decrease after the rigid body movement. After the refinement, the refined structures with *χ*^2^ values less than certain criteria were grouped into a number of clusters according to the root mean square deviations (RMSDs) of Cα atoms of all structure pairs (for example, if 10 structures are used as an input for clustering, 45 (=_10_C_2_) structure pairs exist. Therefore, 45 RMSDs are calculated for 45 structure pairs). Clustering was performed using the gromos method implemented in the GROMACS package^53^. The structures belonging to the first cluster containing the largest number of structures were chosen as the candidate structures (see Supplementary Information for detailed information). More detailed procedures of the ERRB structure refinement aided by MC simulations are described elsewhere^16^.

The structure refinement was performed using each of four difference scattering curves: the difference scattering curves at 0.5 and 1.5 ps, which are labeled as the difference scattering curves of I_0_^U^ and I_0_^D^, and the SADSs of I_0_ and I_1_ (Supplementary Fig. 6b–e). The SADSs of I_0_ and I_1_ indicate the difference scattering curves of I_0_ and I_1_ in their time-independent structures, respectively. The difference scattering curves of I_0_^U^ and I_0_^D^ represent the structural change including the coherent motion with non-thermal origin in addition to the time-independent structure, as the kinetic analysis exhibits a large deviation from the exponential behavior at 0.5 and 1.5 ps. To improve the structure refinement quality, 0.5 and 1.5 ps data that were not subjected to the re-binning were used for the structure refinement because they have higher SNRs than the re-binned data. The contribution of I_1_ to the difference scattering curves at 0.5 and 1.5 ps was subtracted from the difference scattering curves before the structure refinement to extract structural information of the coherent motion of I_0_. For the structure refinement of each curve, 270 initial structures were used. After structure refinement, 108, 100, 95, and 94 candidate structures were obtained for I_0_, I_1_, I_0_^U^, and I_0_^D^, respectively.

Supplementary Methods contain detailed information on sample preparation, structure refinement methods using the WAXS region, the Guinier analysis methods using the SAXS region, and the SAXS region refinement using the WAXS region refinement results.

### Reporting summary

Further information on experimental design is available in the Nature Research Reporting Summary linked to this paper.

## Supplementary information

Supplementary Information

Reporting summary

## Data Availability

Other data that support the findings of this study are available from the corresponding author upon reasonable request. Source data are provided with this paper.

## Source data

Source Data

## Code availability

The code for the ERRB structure refinement aided by MC simulations is available via GitHub repository (https://github.com/yunbeomlee123/ERRB-structure-refinement-aided-by-MC-simulations). Other analysis codes that support the findings of this study are available from the corresponding author upon reasonable request.
