# Supplementary material for: Ultrafast coherent motion and helix rearrangement of homodimeric hemoglobin visualized with femtosecond X-ray solution scattering
Source: Nat Commun. 2021 Jun 16;12:3677. doi: 10.1038/s41467-021-23947-7 (PMC8209046; doi:10.1038/s41467-021-23947-7)
Supplement: Supplementary file 1 — Supplementary Information [file 41467_2021_23947_MOESM1_ESM.pdf]

Supplementary Information for

Ultrafast Coherent Motion and Helix Rearrangement of Homodimeric Hemoglobin Visualized with Femtosecond X-ray Solution Scattering

Yunbeom Lee[1,2,‡], Jong Goo Kim[1,2,‡], Sang Jin Lee[1,2], Srinivasan Muniyappan[1,2], Tae Wu Kim[1,2], Hosung Ki[1,2], Hanui Kim[1,2], Junbeom Jo[1,2], So Ri Yun[1,2], Hyosub Lee[1,2], Kyung Won Lee[1,2], Seong Ok Kim[1,2], Marco Cammarata[3], and Hyotcherl Ihee[1,2]*

*Corresponding author. E-mail: hyotcherl.ihee@kaist.ac.kr

This PDF file includes:

Supplementary Methods
Supplementary Figure 1-16
Supplementary References

Supplementary Methods

Preparation of HbI(CO)$_2$ sample

The HbI sample solution was prepared following the procedures described in our previous publication.[1] A HbI solution with the concentration of ~0.6 mM in 100 mM phosphate buffer (pH 7) was prepared in an air-tight vial sealed with a rubber cap. The concentration of HbI was determined by the absorbance of the sample at 578 nm based on the absorption coefficient of heme-oxygenated derivatives (14.3 mM^{-1} cm^{-1}). The sample was purged with nitrogen gas for

~20 min, and HbI was reduced with 60 μL of 1 M sodium dithionite solution under the nitrogen atmosphere. Then, the reduced sample was exposed to CO gas for ~20 minutes to generate HbI$(CO)_2$. The preparation of the sample solutions was performed just before the x-ray solution scattering measurement.

The effect of the hydration shell changes to the TRXSS signal

In the previous theoretical study on myoglobin (Mb), it was argued that the electron density of the hydration shell around the protein changes during the ultrafast time-domain after reaction initiation triggered by light.[2] We conducted simulations using CRYSOL to verify the effect of electron density change of the hydration shell during the photoreaction on difference scattering curves.[3] In the simulations, three different electron density changes of the hydration shell after photolysis were assumed. For the simulation, a candidate structure of I_1 reported in the previous TRXSS study was selected to generate the difference curves.[1] The difference curves were calculated assuming the transition from the carboxy structure (PDB entry: 3sdh) to the selected candidate structure under three different electron density changes of the hydration shell. The change of the hydration shell density was described by changing the parameter of the contrast of the hydration shell for CRYSOL which uses the implicit solvent model to describe the hydration shell with the thickness of 3 Å. The parameters such as the electron density of the bulk solvent were set as the default value for CRYSOL during the simulations.

According to our simulation results, the time-resolved SAXS signal (especially where $q < 0.1$ Å^{-1}) is affected significantly by the change of electron density of the hydration shell (Supplementary Fig. 1a) regardless of the degree of the electron density changes. Such an effect is absent in the signal of a wider angle region (Supplementary Fig. 1b) in all three electron density changes of the hydration shell. In Supplementary Fig. 1c, the double-difference of theoretical difference curves assuming the different changes of the electron density of the hydration shell (black), and double-differences of the experimental difference curves of the time delays where the oscillatory feature was observed (red and blue) are plotted. The double-difference due to the change of the hydration shell shows different features compared to the double-differences of the experimental data in terms of the shape and amplitude (particularly in the q range from 0.13 Å^{-1} to 0.14 Å^{-1}). In addition, as shown in Supplementary Fig. 9, the changes of R_g and volume during the coherent motion are correlated to the signal amplitude at

q = 0.13 Å^{-1} where the effect of the hydration shell is relatively small. Considering this result, the time-resolved difference scattering signal in the SAXS region with q < 0.13 Å^{-1} was excluded for the kinetic analysis and structure refinement on the fs-WAXS data to exclude the effect due to the hydration shell and elucidate the structural change of the bare protein. To check any dependency on protein structure, the same simulation was performed on other candidate structures of I_1 reported in the previous TRXSS study. The effects of the electron density changes of the hydration shell on the SAXS and WAXS regions were the same regardless of the structures.

In addition, a simulation which considers the change of the reduced form factor was performed to verify the effect of the change of the excluded volume to the difference scattering curves. In this simulation, 'effective atomic radius' which is an input variable for CRYSOL and related to the displaced volume per atomic group was changed to check the effect of the reduced atomic form factor to the difference curve. Default input values for CRYSOL were used for other parameters such as the electron density contrast of the hydration shell. Our structure refinement results indicate that the volume expands less than 1%, which indicates that the average atomic radius would increase less than 0.3%. A candidate structure obtained from the structure refinement was selected and used to calculate the difference scattering curve of the transition from the carboxy structure to the candidate structure, using two different effective atomic radii. The difference scattering curves calculated assuming the original effective atomic radius (1.622) and the effective atomic radius increased by ~0.3% (1.627) show similar features, which indicates that the change of the reduced form factor due to the change of the excluded volume does not change the main interpretation (Supplementary Fig. 1d)

Estimation of instrument response function (IRF) and kinetic analysis

The thickness of the capillary used for the experiment was ~500 μm. The temporal duration of the x-ray pulse was ~50 fs. Other parameters such as the temporal duration of the laser pulse (~250 fs) were obtained from the previous fs-TRXSS study performed at the XPP beamline of LCLS and used for IRF estimation of this study.[4] Considering such parameters and the time jitter between the laser and x-ray pulse, the full-width half maximum (FWHM) of IRF was estimated to be ~800 fs.

The experiment-restrained rigid-body (ERRB) structure refinement aided by Monte Carlo (MC) simulations on fs-WAXS data

Supplementary Fig. 6a shows the change of root-mean-squared-deviations (RMSDs) and χ^2 values after performing the ERRB structure refinement of I_0 as a representative case. The RMSDs and χ^2 values of the initial structures used as starting structures for the structure refinement and the refined structures obtained by the structure refinement are indicated by black and blue dots, respectively. We note that the RMSDs of the initial structures with reference to the carboxy state extend up to ~1.2 Å, which is larger than the RMSD of the crystal structure at 5 ns (~0.16 Å), indicating that the initial structures cover a wider conformational space than the crystal structure at 5 ns can reach. Clustering was performed on the refined structures with χ^2 values less than 250 using the gromos method implemented in the GROMACS package, and the cutoff value for the clustering was 0.045.[5] The structures in the first cluster were selected as the candidate structures indicated by red dots. The mother structures yielding the candidate structures are indicated by magenta dots. The RMSDs between mother structures and candidate structures are about 0.3 Å on average.

Since the number of the fitting parameters is large, the structure refinement using simple MC simulation can induce overfitting. However, ERRB structure refinement aided by MC simulations utilizes a target function in addition to MC simulations to guide the movement of the rigid bodies. Since the target function is composed of various terms such as the χ^2 term and the collision-avoiding term which substantially reduce the effective degrees of freedom and the conformational space, guidance using the target function can decrease the possibility of the overfitting. Indeed, comparison of standard deviations of the RMSDs against the carboxy structure shows that the standard deviation decreases from 0.079 Å for the mother structures of the candidate structures to 0.052 Å for the candidate structures via the structure refinement. This result indicates that the candidate structures from the different mother structures have converged well into a target structure through the guidance of the target function used for the structure refinement.

The calculation of a theoretical difference scattering curves of the reaction intermediates

The theoretical difference scattering curves of the reaction intermediates were calculated by averaging the theoretical difference scattering curves of the candidate structures of the reaction

intermediates. The theoretical difference scattering curves of the reaction intermediates were used to generate the calculated curves shown in Fig. 1b.

The calculation of a difference distance map (DDM)

DDMs were calculated by subtracting the distance map of reference structures from the distance map of target structures. Since the structural changes of two subunits are similar with the RMSD of ~0.3 Å (Supplementary Fig. 8a), the distance of the same residue pairs in each subunit of HbI were averaged to calculate intra-subunit distance maps. For instance, the distance between residue *i* and residue *j* in subunit A was averaged with the distance between residue *i* and residue *j* in subunit B. For inter-subunit distance map calculation, a distance between a residue pair in subunit A and subunit B (for example, residue *i* in subunit A and residue *j* in subunit B) was averaged with the distance between the same residue pair but in subunit B and subunit A (residue *j* in subunit A and residue *i* in subunit B). For calculating DDMs using I_0 as a reference structure, we used the averaged distance maps of candidate structures of I_0.

Similarity between difference distance maps (DDMs)

Similarity between two difference distance maps was calculated using Pearson correlation coefficient. Pearson correlation coefficient between DDMs was calculated using the components in the DDMs as follows.

$$R = \frac{\sum_{i,j}(x_{i,j}-\bar{x})(y_{i,j}-\bar{y})}{\sqrt{[\sum_{i,j}(x_{i,j}-\bar{x})^2][\sum_{i,j}(y_{i,j}-\bar{y})^2]}} \quad (1)$$

, where $x_{i,j}$ and $y_{i,j}$ are the (*i*, *j*)th components of the region of interest of two DDMs, and $\bar{x}$ and $\bar{y}$ are the means of $x_{i,j}$ and $y_{i,j}$, respectively.

The calculation of a displacement plot

The displacement was calculated as the changes in the distance of the Cα atom of each residue from the iron atom with respect to the reference structure. In the case of HbI, which has a

homodimeric structure, the displacements of the same residue in each subunit were averaged to generate the displacement plot of a candidate structure. Finally, the displacement plots of the candidate structures were averaged to yield the averaged displacement plot of the reaction intermediate.

The calculation of the radius of gyration (R_g) and volume for fs-WAXS data

R_g was calculated from the candidate structures of the structure refinement results using the following equation,

$$R_g = \sqrt{\frac{\sum_i m_i (r_i - r_{\mathrm{COM}})}{\sum_i m_i}} \tag{2}$$

, where m_i and r_i are the mass and the position of the atoms or atomic groups in the protein, respectively, and r_{COM} is the position of the center of the mass of the protein. The volumes of the protein structures were calculated using the volume calculation program package developed and distributed by Voss *et al*.[6] The errors of R_g and volume were calculated as the standard error of the mean (SEM) among the candidate structures.

Time profile calculation of the radius of gyration (R_g) and volume for fs-WAXS data

For the WAXS data, the changes of the radius of gyration (ΔR_g) and volume (ΔV) with respect to the carboxy structure (PDB entry: 3sdh) at a time delay were calculated by adding ΔR_g and ΔV of two reaction intermediates (I_0 and I_1) multiplied by their concentrations using the following equations.

$$\Delta R_g{}^{\mathrm{WAXS}}(t) = \Delta R_g{}^{\mathrm{I}_0} \times C^{\mathrm{I}_0}(t) + \Delta R_g{}^{\mathrm{I}_1} \times C^{\mathrm{I}_1}(t) \tag{3}$$

$$\Delta V^{\mathrm{WAXS}}(t) = \Delta V^{\mathrm{I}_0} \times C^{\mathrm{I}_0}(t) + \Delta V^{\mathrm{I}_1} \times C^{\mathrm{I}_1}(t) \tag{4}$$

where $\Delta R_g{}^{X}$ and ΔV^{X} are ΔR_g and ΔV of the reaction intermediate X, respectively, and $C^{X}(t)$ is the concentration of the reaction intermediate X at the time delay, t. For 0.5 and 1.5 ps, ΔR_g and ΔV obtained from the candidate structures of $I_0{}^{U}$ and $I_0{}^{D}$ were used for the calculation. For the time delays earlier than -0.3 ps, ΔR_g and ΔV were assumed to be zero. The error was estimated with the SEMs of ΔR_g and ΔV of the reaction intermediates. The time profiles of the

ΔR_g^{WAXS} and ΔV^{WAXS} calculated using Supplementary Equations (3) and (4) are shown in Fig. 4a. Since the time profiles of ΔR_g^{WAXS} and ΔV^{WAXS} were calculated by taking linear combinations of ΔR_g and ΔV of two reaction intermediates, the errors for the time profiles were calculated using the propagation of the error upon the linear combination.

The Guinier analysis of fs-SAXS data

The Guinier analysis of the fs-SAXS data was performed following a previously reported method.[4] The difference scattering curve at each time delay was fitted using the following equation.

$$\Delta I(q,t) = C(t) \times \{I(q,V(t),R_g(t)) - I_{\text{carboxy}}(q,V_{carboxy},R_{g,carboxy})\} \quad (5)$$

, where $I(q,V,R_g)$ is the intensity calculated using the following Guinier approximation and $C(t)$ is the population of the photolyzed HbI.

$$I(q,V,R_g) = A \times I_{q=0} e^{(-R_g^2 q^2/3)} = A(\rho_p V - \rho_b V) e^{(-R_g^2 q^2/3)} \quad (6)$$

In Supplementary Equation (6), A is a scaling factor between the observed and theoretical intensities, V is the volume of the protein, and ρ_p and ρ_b are the electron densities of the protein and the solvent, respectively. For consistency, we used the same scaling factor (A) used in the ERRB structure refinement aided by MC simulations for the Guinier analysis. Other parameters used for the fitting were $\rho_p V$ = 17740 (electron), ρ_b = 0.34 (electron/Å^3). The R_g and volume of the carboxy structure were calculated following the aforementioned methods used for the analysis of fs-WAXS data. The fitting and error analysis were conducted using the MINUIT package written at CERN.[7] The changes of R_g and volume of the system were calculated by $C(t)R_g(t)$ and $C(t)V(t)$. The changes of R_g and volume at time delays earlier than -0.3 ps were assumed to be zero, since the changes of R_g and volume were inaccurate at these time delays due to the negligible amount of the photolyzed HbI.

The fitting of the SAXS region using the change of electron density of the hydration shell

The fitting was performed in a similar way to that described in Fig. 1b. The calculated curve at each time delay was obtained by a linear combination of the theoretical difference scattering

curves of the reaction intermediates (I_0, I_1, I_0^U, and I_0^D). The theoretical difference scattering curve of each reaction intermediate was calculated by averaging the theoretical difference scattering curves of the candidate structures. The electron density of the hydration shell was modified during the calculation of the theoretical scattering curves of the candidate structures. The calculation of the scattering curve was performed using CRYSOL. The coefficient for the linear combination was determined by the concentration of each reaction intermediate at each time delay. The fitting was performed so that the difference between the calculated curve and the experimental difference scattering curve at each time delay was minimized. The changes of the electron density of the hydration shell at time delays earlier than -0.3 ps were assumed to be zero due to the negligible amount of the photolyzed HbI at these time delays.

Single-layer model vs. multiple-layer model to describe the density change in the hydration shell

In a previous theoretical study on Mb, it was suggested that the solvent molecules located farther than 3 Å from the protein can be perturbed due to the pressure generated by the protein quake.[2] Since CRYSOL, the program used to calculate the scattering curve, describes the hydration shell as a layer with a thickness of 3 Å, the density change dependent on the distance from the protein surface is approximated as a constant density of a single hydration shell instead of the density changes of multiple shell layers. To check the effect of this approximation, we performed a series of simulations considering the effect of multiple shell layers and their density changes.

In the simulations, the protein body and solvent layers were assumed a sphere with a radius of 21 Å and spherical shells with a thickness of 3 Å, respectively. The form factor for the protein body was calculated as follows.

$$f_{\text{protein}}(q,R) = \Delta e_{\text{protein}} \times f_{\text{sphere}}(q,R) = \Delta e_{\text{protein}} \frac{3[\sin(qR) - qR\cos(qR)]}{(qR)^3} \quad (7)$$

, where $\Delta e_{\text{protein}}$ is the difference between the number of electrons in the protein and that in the bulk solvent and R is the radius of the protein body. $\Delta e_{\text{protein}}$ was determined using the volume of the protein and the electron densities of the protein (0.430 $e^-/Å^3$) and the bulk solvent (0.334 $e^-/Å^3$). The form factor for the solvent layers were calculated as follows.

$$
\begin{aligned}
f_{\text{layer}}(q, R_{\text{outer}}, R_{\text{inner}}) &= \Delta e_{\text{layer}} \times f_{\text{spherical shell}}(q, R_{\text{outer}}, R_{\text{inner}}) \\
&= \Delta e_{\text{layer}} \frac{V(R_{\text{outer}}) f_{\text{sphere}}(q, R_{\text{outer}}) - V(R_{\text{inner}}) f_{\text{sphere}}(q, R_{\text{inner}})}{V(R_{\text{outer}}) - V(R_{\text{inner}})}
\end{aligned} \tag{8}
$$

, where Δe_{layer} is the difference between the number of electrons in the solvent layer and that in the bulk solvent, R_{outer} and R_{inner} are the outer and inner radius of the shell, and $V(R_{\text{outer}})$ and $V(R_{\text{inner}})$ are the volumes of the spheres with radius of R_{outer} and R_{inner}, respectively. Δe_{layer} was determined using the volume of the water layer and the electron densities of the water layers (0.364 $e^-/Å^3$ for the 1st layer and 0.334 $e^-/Å^3$ for the other layers) and the bulk solvent (0.334 $e^-/Å^3$). The x-ray scattering intensity of the protein with n solvent layers were calculated by taking square of the summed form factors as follows.

$$
I(q) = [f_{\text{protein}}(q) + \sum_{n} f_{i\text{-th layer}}(q)]^2 \tag{9}
$$

To investigate how the simplified single-layer description for the hydration shell used in CRYSOL can affect the resulting electron density of hydration layer compared to the result obtained from the multiple-layer description, we compared the difference curve generated from the change of the two solvent layers (multiple-layer model) with that generated from the change of the 1st solvent layer (single-layer model), as depicted in Supplementary Fig. 12a. The difference curves of two models were calculated by subtracting the x-ray scattering intensity before the electron density change of the layer from that after the electron density change. The difference curves of two models show agreement as shown in Supplementary Fig. 12b when the net changes of the number of electrons of the two models were set to be the same. This result indicates that the overall change of electron densities in the multiple solvent layers can be approximated using the single-layer model.

In addition, we performed another simulation to check how the contribution of electron density change in the solvent layers to the x-ray scattering signal depends on the distance of the solvent layers from the protein body. To calculate the effect of the electron density change of the i-th solvent layer to the difference curve, only the electron density of the i-th water layer was changed as depicted in Supplementary Fig. 12c. Then, the difference curve was calculated by subtracting the x-ray scattering curves before and after the density change. The magnitude of the difference curve due to the electron density change in the i-th water layer was quantified by taking the squared sum of the difference scattering intensities within the q range from 0.05 $Å^{-1}$ to 0.1 $Å^{-1}$ and normalized by that of the 1st solvent layer (that is, the relative contribution

to the signal). The simulation result shown in Supplementary Figure 12d indicates that the electron density changes in the solvent layers closer to the protein body have higher contributions to the difference curve.

Kinetic and structural analysis of myoglobin (Mb)

The collection of TRXSS data of Mb was described elsewhere.[4] The TRXSS data of Mb was processed and analyzed in a similar way to that used for the case of HbI. The data with $0.17\ \text{Å}^{-1} \leq q \leq 1.0\ \text{Å}^{-1}$ was used for the WAXS analysis. The kinetic analysis was performed via SVD analysis and kinetic modeling. The SVD analysis was performed in a similar way to that used for HbI. From the SVD results, two SVD components were classified as the major components. The rSVs were reconstructed with two exponential decay functions convoluted with an IRF (500 fs). One of the time constants used in the rSV reconstruction was fixed to 70 ps during the analysis, which is the time constant observed in the previous TRXSS study on horse heart Mb.[8] As a result of the SVD analysis, the rSVs were reconstructed with two exponential decay functions with time constants of 3.2 and 70 ps. Based on the SVD analysis results, kinetic modeling was performed using a sequential kinetic model in a similar manner used for the case of HbI.

ERRB structure refinement aided by MC simulations was conducted against the SADSs obtained from the kinetic modeling to elucidate the structures of intermediates. Crystal structures of horse heart Mb at 500 fs (PDB entry: 5cnb) and 10 ps (PDB entry: 5cne) after photolysis, which were obtained from a TR-SFX study on Mb, were used as the template structures for the 1st and 2nd intermediate, respectively. The carboxy structure of Mb obtained from the same TR-SFX experiment (PDB entry: 5cmv) was used to calculate the theoretical difference scattering curve during the structure refinement. During structure refinement, the structure of Mb was regarded as composed of nine rigid bodies according to its eight helices and a heme group. After structure refinement, 76 and 71 candidate structures obtained for the 1st and 2nd reaction intermediate, respectively. The global structural change of Mb was analyzed using R_gs and volumes of the intermediates. R_gs and volumes of the intermediates were calculated by averaging the R_gs and volumes of the candidate structure of the intermediates. The R_g and volume of each candidate structure were calculated following the aforementioned methods used to analyze the fs-WAXS data.

Supplementary figures

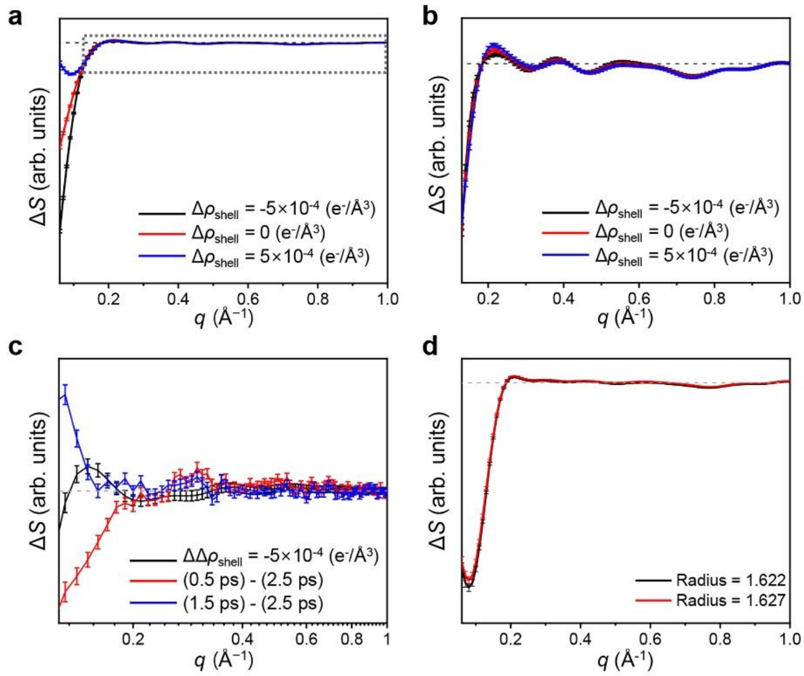

Supplementary Fig. 1. (a-c) The simulation results on the effect of the electron density change in the hydration shell on difference scattering curves. To verify the effect of the electron density change of the hydration shell during the photoinduced structural change from HbI(CO)$_2$ to I$_1$ on difference scattering curves, three different electron density changes were used to generate difference scattering curves. Difference scattering curves with three different electron density changes of hydration shells are plotted for (a) SAXS and WAXS regions ($0.06 \leq q \leq 1.0$ (Å^{-1})) and (b) only the WAXS region ($0.13 \leq q \leq 1.0$ (Å^{-1})). The WAXS region shown in (b) is on the same scale as that marked by gray dots in (a). (c) The double-difference of theoretical difference curves assuming the different changes of the electron density of the hydration shell (black), and double-differences of the experimental difference curves of the time delays where the oscillatory feature was observed (red and blue). (d) The simulation results on the effect of the change of the excluded solvent volume to the difference scattering curves. The difference scattering curves assuming two different effective atomic radii are plotted for comparison. The experimental SEM at 100 ps was used to estimate the SEM of difference scattering curves for all difference and double-difference curves.

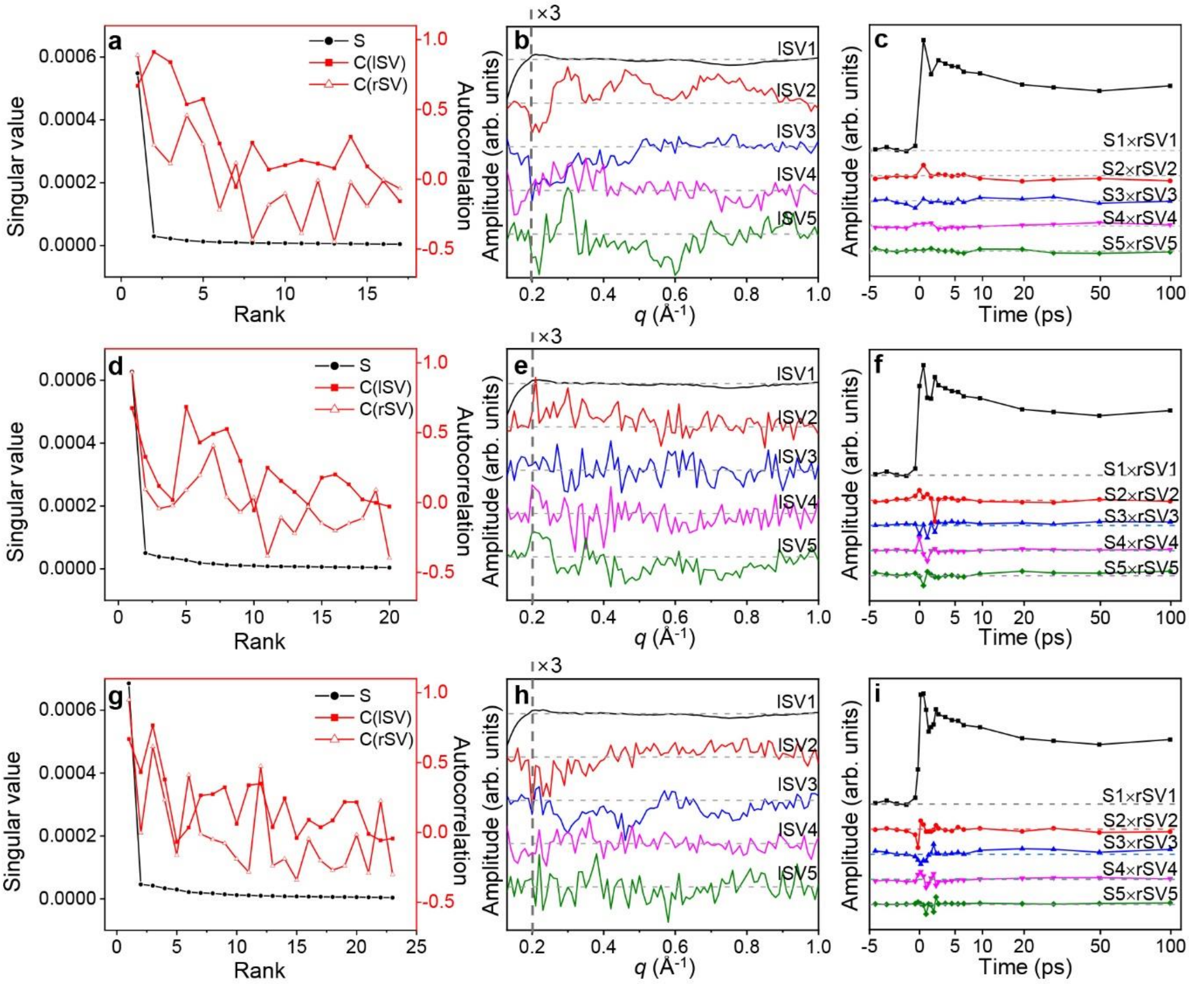

Supplementary Fig. 2. Singular value decomposition (SVD) results of fs-WAXS data ($0.13 \leq q \leq 1.0$ (Å^{-1})) of HbI(CO)$_2$ for (a-c) 1-ps-binned data, (d-f) 500-fs -binned data, and (g-i) 333-fs-binned data. (a, d, and g) Singular values (*S*s) (black filled circle), autocorrelations of left singular vectors (lSVs) (red filled square), and autocorrelation of the right singular vectors (rSVs) (red open triangle) for (a) 1-ps-binned data, (d) 500-fs-binned data, and (g) 333-fs-binned data. (b, e, and h) The first five lSVs are plotted for (b) 1-ps-binned data, (e) 500-fs-binned data, and (h) 333-fs-binned data, respectively. For clarity, the high *q* region of lSVs with $q \geq 0.2$ (Å^{-1}) is scaled up by 3. (c, f, and i) The first five rSVs are plotted for (c) 1-ps-binned data, (f) 500-fs-binned data, and (i) 333-fs-binned data, respectively. The rSVs are scaled by their *S*s to illustrate their contribution to the experimental signal.

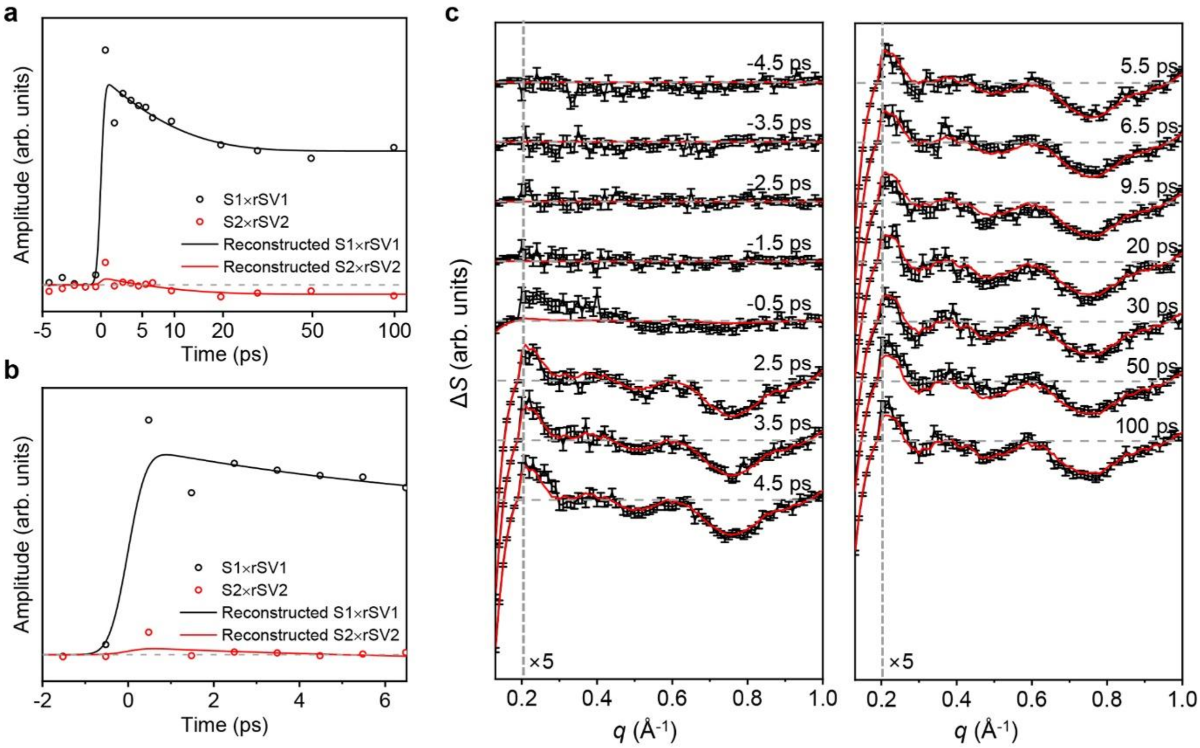

Supplementary Fig. 3. (a, b) The reconstruction results of the 1st and 2nd rSVs in (a) the full and (b) early time-domains (< 6.5 ps). The 1st rSV (black) and 2nd rSV (red) were reconstructed with a single exponential with a time constant of 8.7 (± 0.6) ps. The points at 0.5 and 1.5 ps were excluded from the reconstruction because they showed obvious deviations from the exponential behavior. The rSVs scaled by their singular values (*S*s) were reconstructed instead of rSVs themselves to consider the contributions of rSVs to the experimental data. (c) The results of the kinetic modeling. A sequential model in which I_0 is formed within the instrument response function and transforms into I_1 with a time constant of 8.7 ps was used. The experimental curves were fit using the linear combination of the 1st and 2nd lSVs and the concentration profiles of I_0 and I_1 obtained based on the sequential kinetic model. For comparison, the fitting results (red) are overlaid with the experimental curves (black). The data at 0.5 and 1.5 ps, which show large deviations from the exponential behavior, are excluded from fitting. For clarity, the high q region with $q \geq 0.2$ (Å^{-1}) is scaled up by 5.

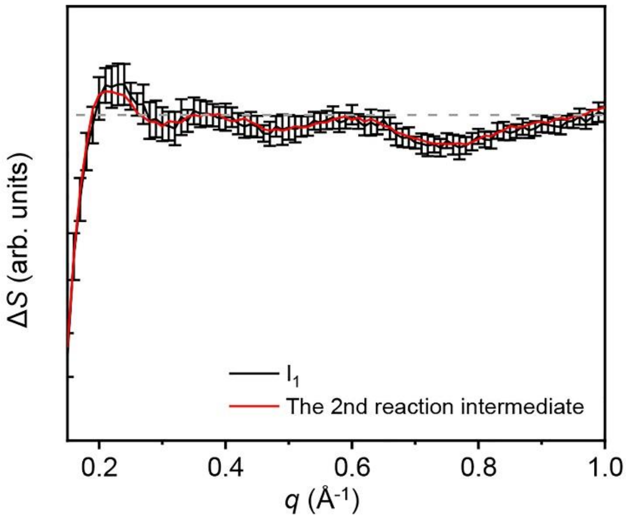

Supplementary Fig. 4. The species-associated difference scattering curve (SADS) of I_1 and its SEM reported in the previous TRXSS study (black) and that of the 2nd reaction intermediate obtained in this study (red).[1] While the SADS of this work has a much better SNR than that of the previous work, the agreement of two curves confirms that the 2nd intermediate in this work corresponds to I_1.

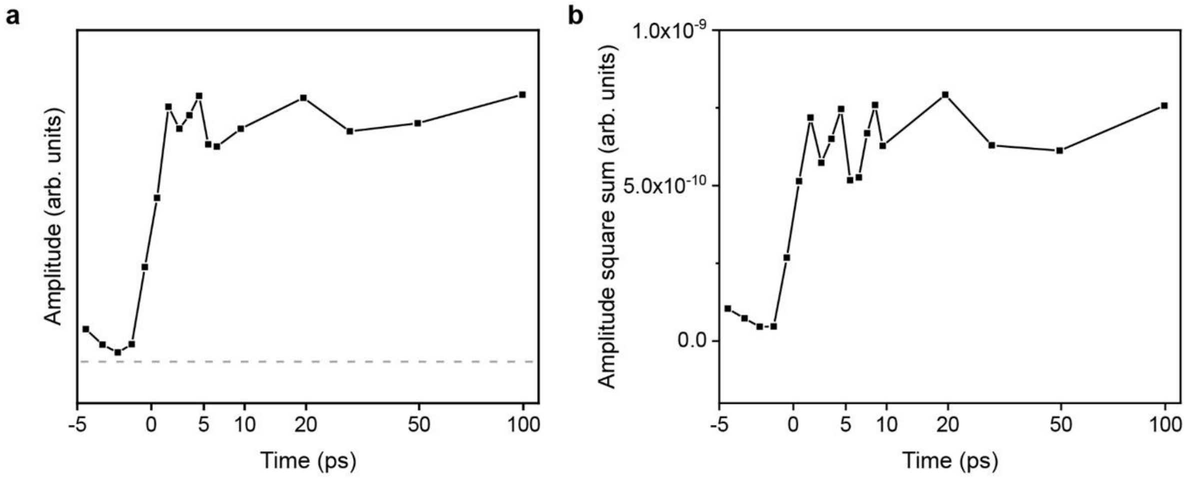

Supplementary Fig. 5. (a) The 1st rSV of SVD results from SVD performed on fs-WAXS data with the high q range from 0.2 to 1.0 Å^{-1}. The 1st rSV of SVD results using the high q region does not exhibit oscillatory behavior, which indicates that the oscillatory behavior observed in the 1st rSV of SVD results using the full WAXS data ($0.13 \leq q \leq 1.0$ Å^{-1}), which is shown in Fig. 1d, originates from the small q range ($0.13 \leq q \leq 0.2$ Å^{-1}) of the WAXS data. (b) The squared sum of the amplitude of the difference scattering curves as a function of time delays. The intensity of the difference scattering curve in the q range of $0.2 \leq q \leq 1.0$ (Å^{-1}) was squared and summed for each time delay to represent the overall signal amplitude of the difference scattering curves. The squared sum of the amplitude remains at a similar level without a clear increase or decrease after the rise of the signal is completed. This feature indicates that neither the structural change due to the cooperativity nor the geminate recombination was observed in the data in the q range of $0.2 \leq q \leq 1.0$ (Å^{-1}). If such phenomena occurred, they should accompany the increase and the decrease of the amplitude of the difference scattering curves, since the cooperativity would increase the number of subunits undergoing the photoreaction and the geminate recombination would decrease the number of photolyzed protein molecules, respectively (See the text for the details). The data of the q range of $0.13 \leq q < 0.2$ (Å^{-1}), where the signal is affected by the coherent motion of the protein (See Supplementary Figs. 2c, 5a), was excluded from the summation.

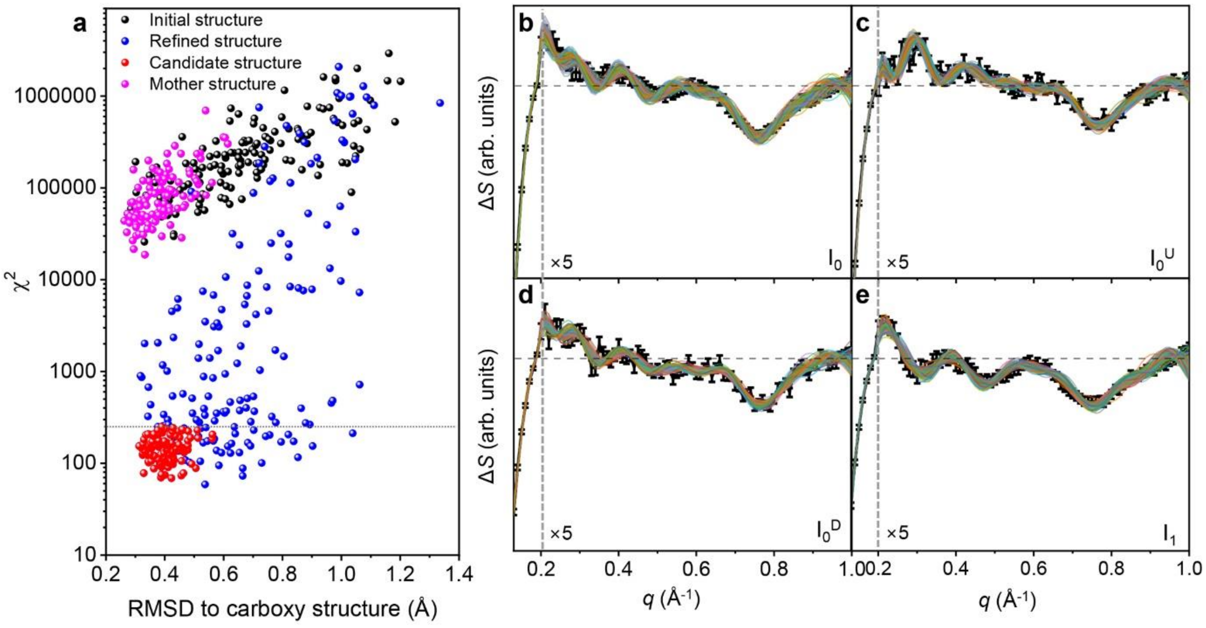

Supplementary Fig. 6. (a) The results of the ERRB structure refinement aided by MC simulations on I_0. The root-mean-square-deviation (RMSD) against the carboxy structure and χ^2 of the initial structures of the refinement (black dots) and the refined structures (blue dots) are plotted. The refined structures with χ^2 values less than 250 marked by the dashed line were selected and clustered according to the RMDSs of Cα atoms of all structure pairs. The structures belonging to the cluster containing the largest number of structures were chosen as the candidate structures indicated by red dots. The mother structures of the candidate structures are indicated by magenta dots. (b-e) Structure refinement results of (b) the SADS of I_0, (c) the data at 0.5 ps (I_0^U), and (d) 1.5 ps (I_0^D), and (e) the SADS of I_1. The target difference scattering curves such as the SADSs and experimental time-resolved difference scattering curves with their SEMs are plotted in black, and the theoretical difference scattering curves of the candidate structures are plotted in other colors. For clarity, all panels are on the same scale, and the high q region with $q \geq 0.2$ (Å^{-1}) is scaled up by 5.

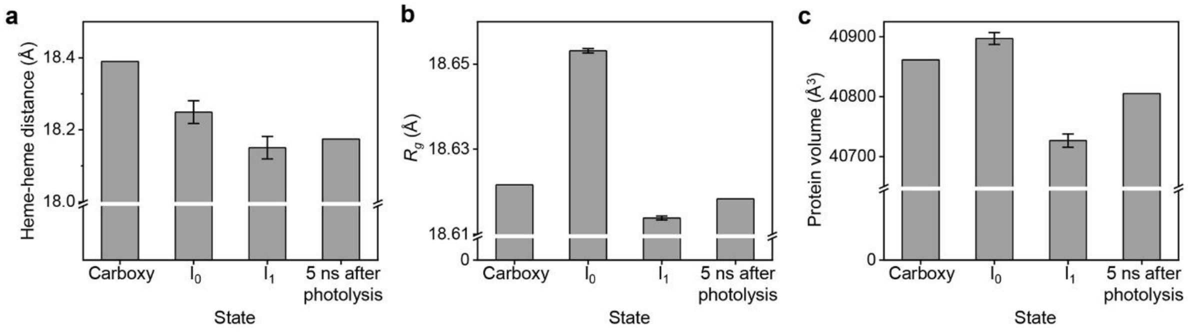

Supplementary Fig. 7. (a) The heme-heme distances, (b) radius of gyration (R_g), and (c) protein volume of the carboxy structure (PDB entry: 3sdh), I_0, I_1, and the crystal structure at 5 ns after photolysis (PDB entry: 2grz). Data are represented as mean values ± SEM. The heme-heme distance was calculated as the distance between the average positions of four nitrogen atoms of each heme group. For I_0 and I_1, the heme-heme distance, R_g, and protein volume were averaged over the candidate structures of the reaction intermediates. The number of candidate structures used for the analysis were 108 and 100 for I_0 and I_1, respectively.

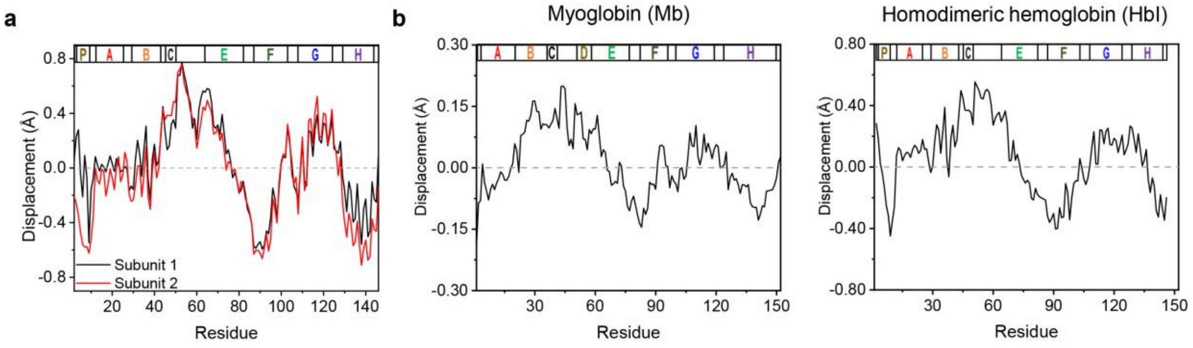

Supplementary Fig. 8. (a) Comparison of the displacement plot of two subunits of a candidate structure of I_0. (b) Comparison of the averaged displacement plot of Mb and HbI with respect to the carboxy structures. For Mb, the crystal structure at 0.5 ps after photoexcitation (PDB entry: 5cnb), which was used to represent the initially-formed intermediate, and the carboxy structure retrieved in the same experiment (PDB entry: 5cmv) was used for the displacement calculation. For HbI, the candidate structures of I_0 and the carboxy structure (PDB code: 3sdh) were used for displacement calculation to represent the initially-formed reaction intermediate. The displacements show that Mb and HbI have similar patterns of displacements. The helices are labeled at the top of each panel. The helix label follows the helix identification in the carboxy structure of Mb and HbI (PDB entry: 5cmv for Mb and 3sdh for HbI).

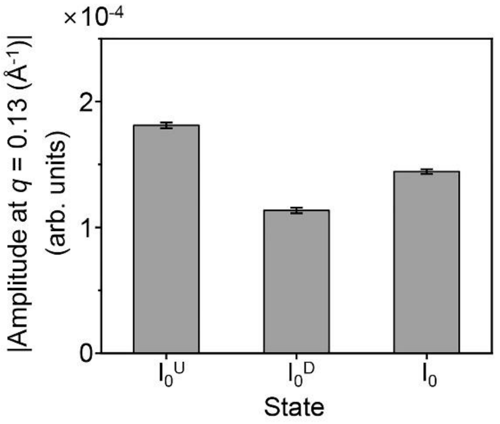

Supplementary Fig. 9. The change of the absolute amplitudes of the difference scattering signal. The absolute amplitude at q = 0.13 Å^{-1} of I_0, I_0^U, and I_0^D. Data are presented as mean values ± SEM. The amplitudes of I_0^U and I_0^D were corrected to exclude the contribution of I_1 to the experimental data. The number of candidate structures used for the analysis were 108, 95, and 94 for I_0, I_0^U, and I_0^D, respectively.

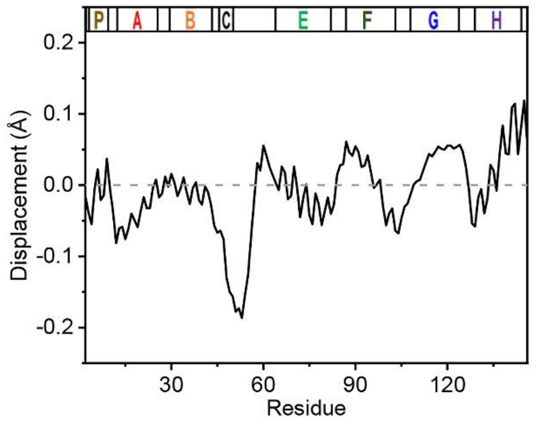

Supplementary Fig. 10. Averaged displacement change of the iron atom of the heme group from I_1 to I_0. The latter was used as the reference. The helices are labeled at the top of the figure. The helix label follows the helix identification in the carboxy structure of HbI (PDB entry: 3sdh).

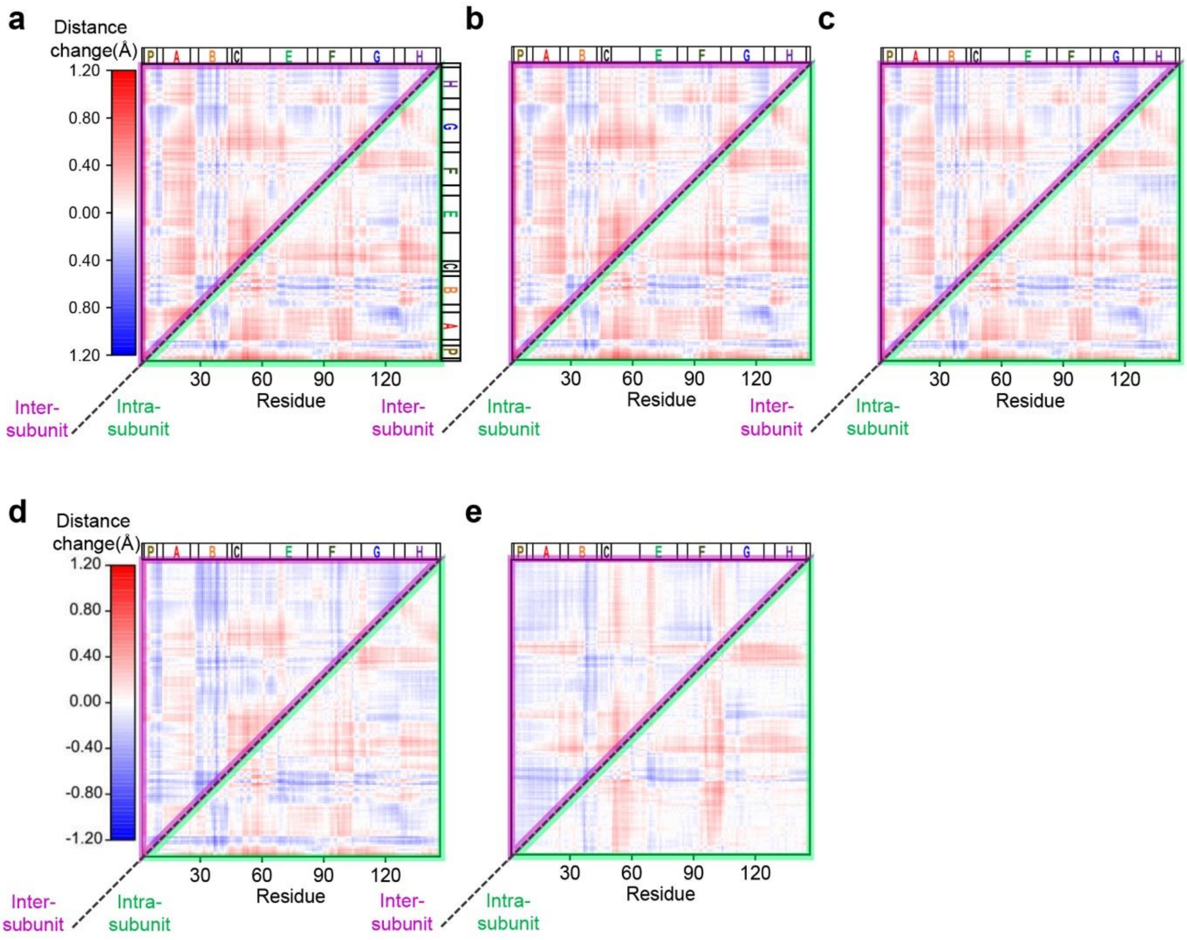

Supplementary Fig. 11. Averaged difference distance maps of (a) I_0, (b) I_0^U, (c) I_0^D, and (d) I_1. (e) Difference distance maps of the crystal structure (PDB entry: 2grz), which is a crystal structure at 5 ns after photolysis. For every case, the carboxy structure was used as the reference structure. For each panel, the upper triangle marked with the magenta line is the inter-subunit difference distance map, and the lower triangle marked with the green line is the intra-subunit difference distance map. All difference distance maps are plotted with the same color gradient shown in (a) and (d). The helices are labeled at the top of each panel. The helix label follows the helix identification in the carboxy structure of HbI (PDB entry: 3sdh).

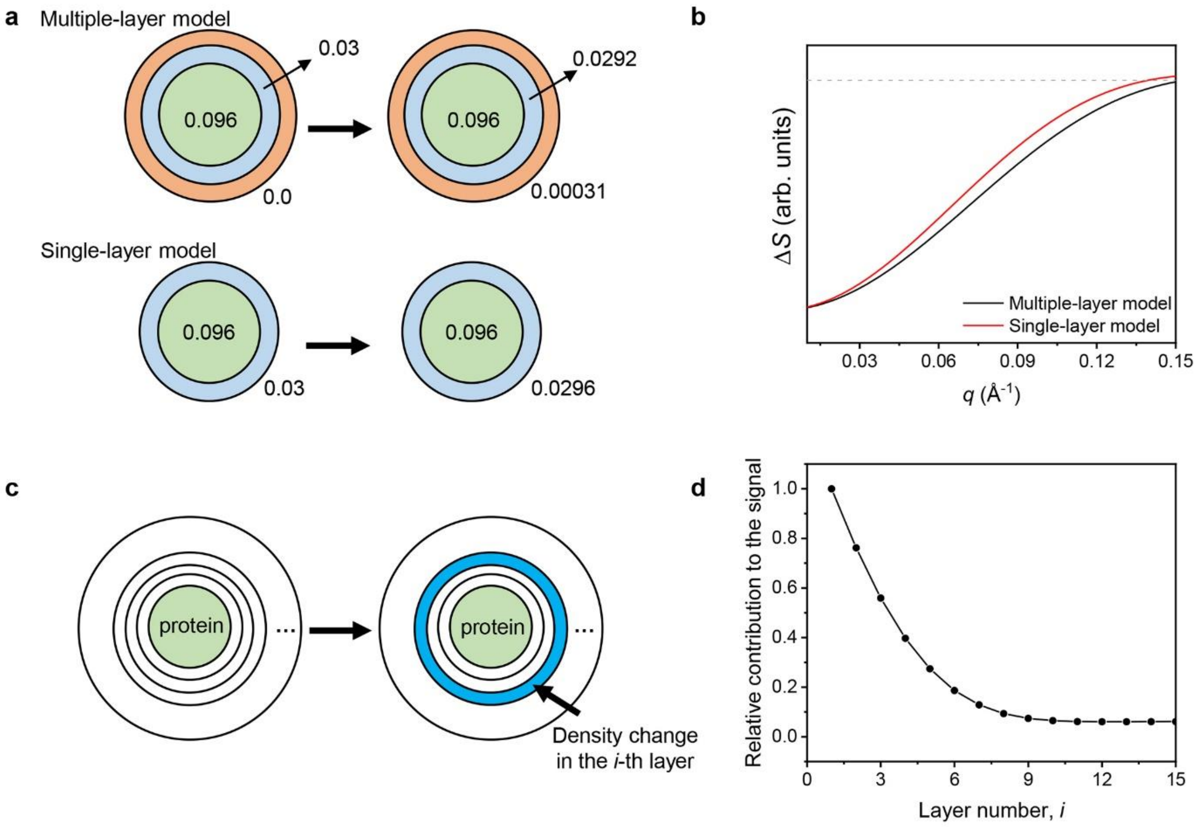

Supplementary Fig. 12. (a, b) The simulation results for describing the electron density change of the multiple solvent layers using the single-layer model. (a) In the multiple-layer model, the electron densities of two solvent layers with a thickness of 3 Å were changed. In the single-layer model, only the electron density of the layer with a thickness of 3 Å was changed. (b) The difference curves obtained using the multiple-layer model (black) and the single-layer model (red). (c, d) The simulation results for the effects of the distance of the solvent layer from the protein. (c) To calculate the contribution of the electron density change of the *i*-th solvent layer to the difference curve, only the electron density of the *i*-th solvent layer was changed and those of the other layers were fixed to their original densities. (d) The relative contribution of electron density changes of the solvent layers to the signal according the layer number (*i*).

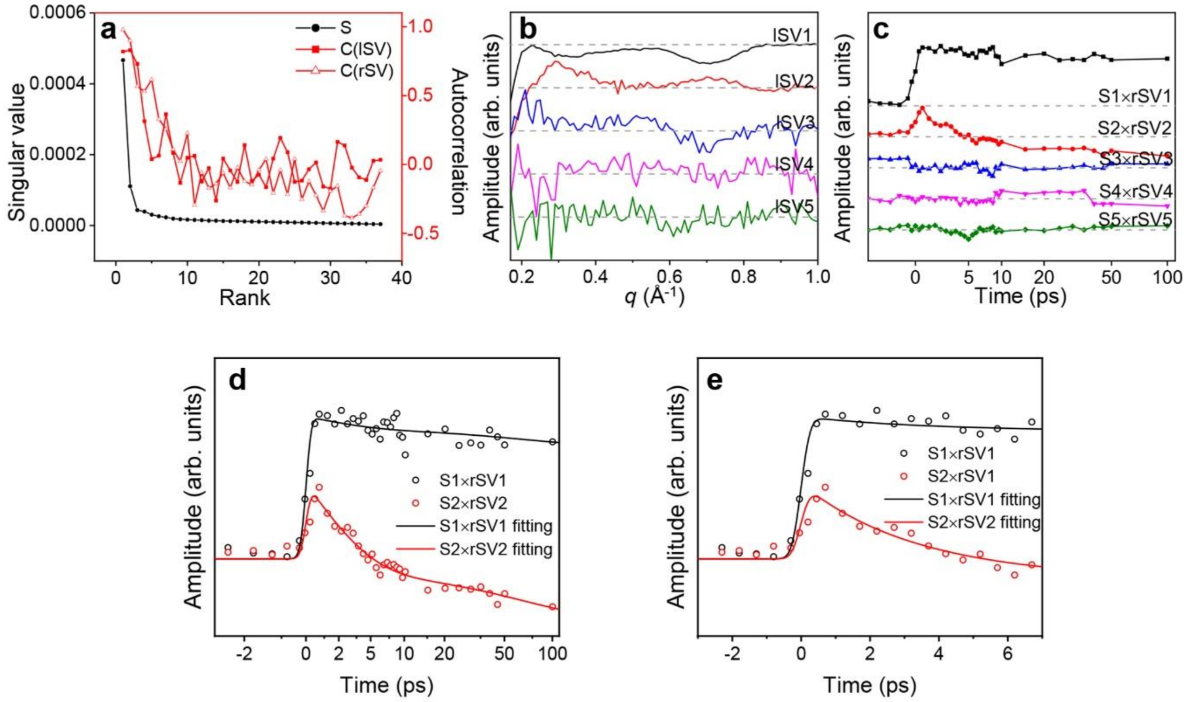

Supplementary Fig. 13. Singular value decomposition (SVD) and kinetic fit results of fs-WAXS data ($0.17 \leq q \leq 1.0$ ($Å^{-1}$)) of MbCO. (a) Singular values (*S*s) (black filled circle) and autocorrelations of lSVs (red filled square) and rSVs (red open triangle) are plotted. The first five lSVs and rSVs are plotted in (b) and (c), respectively. The rSVs are scaled by their *S*s to illustrate their contribution to the experimental signal. The first two singular vectors were selected as the major components. Exponential fit results of the 1st rSV (black) and 2nd rSV (red) in the full and early time-domains (< 7 ps) are plotted in (d) and (e), respectively. The 1st and 2nd rSVs scaled by their *S*s are reconstructed with two exponential decay functions and do not exhibit oscillatory features.

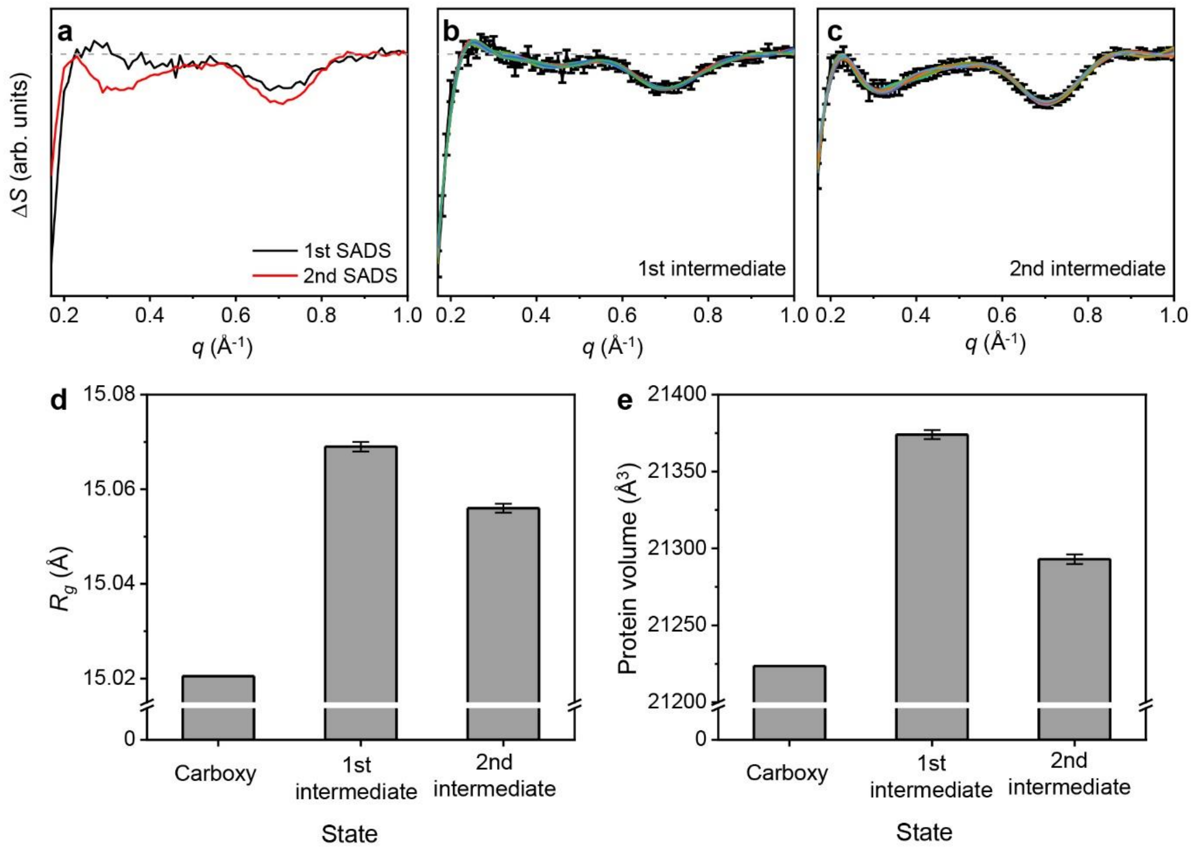

Supplementary Fig. 14. (a) The species-associated difference scattering curves (SADSs) of the two reaction intermediates involved in the ultrafast structural dynamics of Mb and (b, c) comparison of SADSs and the theoretical difference scattering curves obtained from the structure refinement. The SADSs and their SEM are plotted in black, and the theoretical difference scattering curves of the candidate structures are plotted in other colors for (b) and (c). All panels are plotted on the same scale. (d, e) The (d) radius of gyration (R_g) and (e) protein volume of the carboxy structure and the reaction intermediates of Mb. For the reaction intermediates, the R_g and protein volume were averaged over the candidate structures. Error bars represent SEM. The number of the candidate structure for the analysis were 76 and 71 for the 1st and 2nd intermediate, respectively.

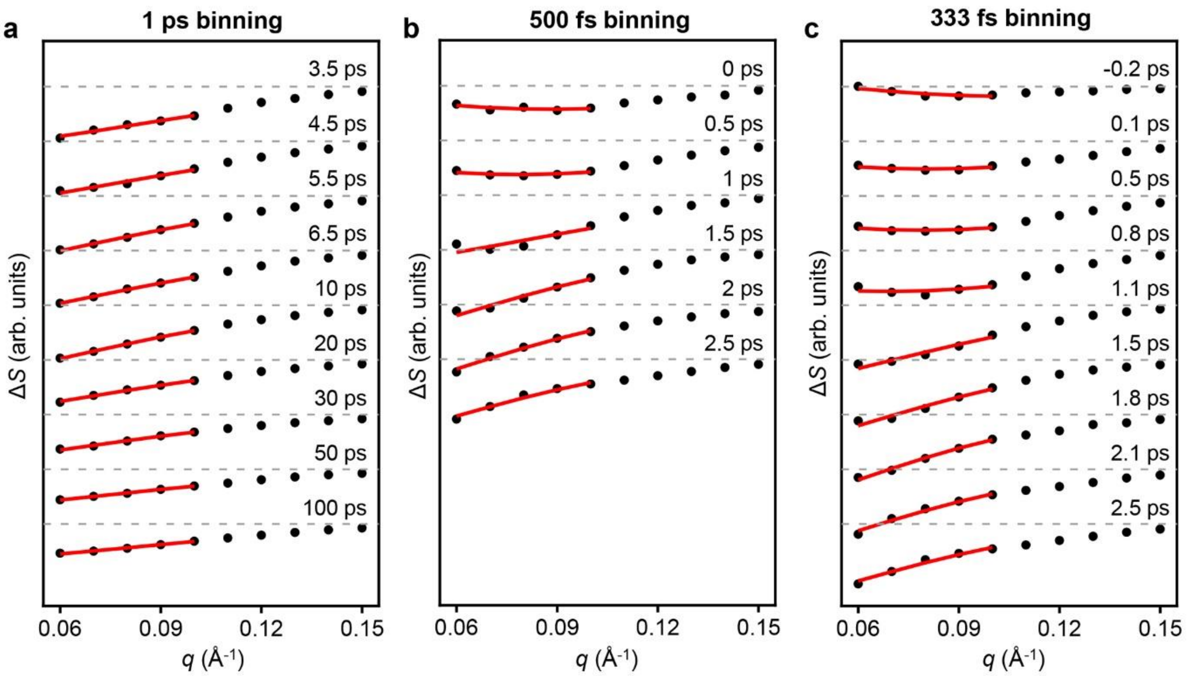

Supplementary Fig. 15. The results of the Guinier analysis on the SAXS region of TRXSS data ($0.06 \leq q \leq 0.1$ (Å^{-1})) of HbI. The experimental data and the fitted results of (a) 1-ps-binned data, (b) 500-fs-binned data, and (c) 333-fs-binned data are plotted in black circles and red lines, respectively.

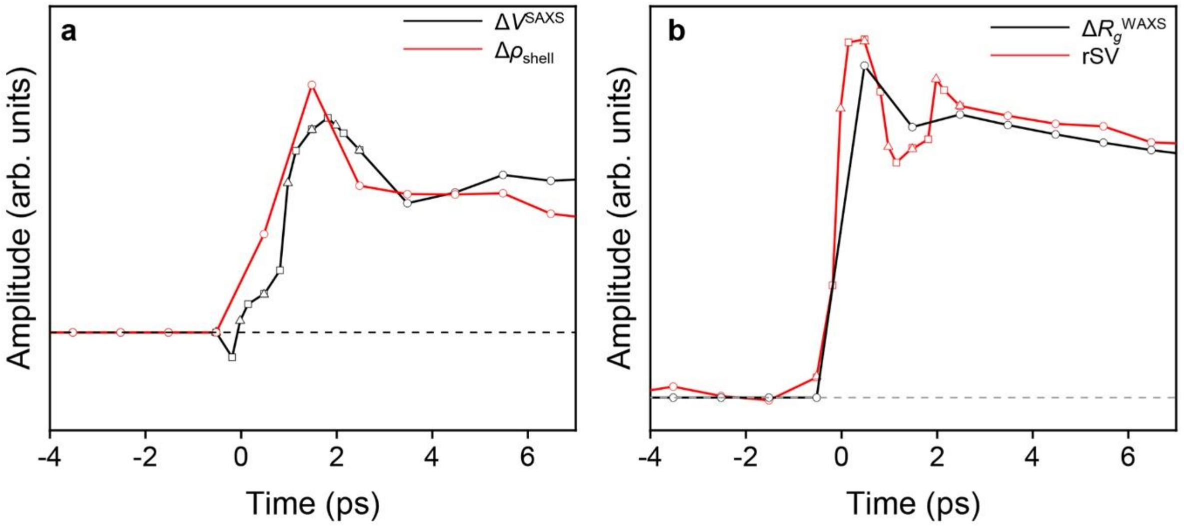

Supplementary Fig. 16. (a) Comparison of the time profile of the ΔV^{SAXS} (black) and the change of electron density of the hydration shell ($\Delta\rho_{shell}$, red) in the early time-domain (< 7 ps). The results from the 333-fs-binned data, 500-fs-binned data, and 1-ps-binned data are plotted with square, triangle, and circle, respectively. A negative scaling factor was multiplied to $\Delta\rho_{shell}$ for comparison because $\Delta\rho_{shell}$ is negative in the ultrafast time-domain whereas ΔV^{SAXS} is positive. (b) Comparison of the time profile of the ΔR_g^{WAXS} (black) and 1st rSV (red) of HbI in the early time-domain (< 7 ps). The results from the 333-fs-binned data, 500-fs-binned data, and 1-ps-binned data are plotted with square, triangle, and circle, respectively. Because ΔR_g^{WAXS} has a negative asymptotic value whereas the 1st rSV has a positive asymptotic value, an offset was added after time zero during ΔR_g^{WAXS} profile calculation for comparison. Such an addition does not alter the overall oscillatory feature.

Supplementary References

1. Kim, K. H., *et al.* Direct observation of cooperative protein structural dynamics of homodimeric hemoglobin from 100 ps to 10 ms with pump-probe X-ray solution scattering. *J. Am. Chem. Soc.* **134,** 7001-7008 (2012).
2. Brinkmann, L. U. L., Hub, J. S. Ultrafast anisotropic protein quake propagation after CO photodissociation in myoglobin. *Proc. Natl. Acad. Sci. USA* **113,** 10565-10570 (2016).
3. Svergun, D., Barberato, C., Koch, M. H. J. CRYSOL - A program to evaluate x-ray solution scattering of biological macromolecules from atomic coordinates. *J. Appl. Crystallogr.* **28,** 768-773 (1995).
4. Levantino, M., *et al.* Ultrafast myoglobin structural dynamics observed with an X-ray free-electron laser. *Nat. Commun.* **6,** 6772 (2015).
5. Berendsen, H. J. C., Vanderspoel, D., Vandrunen, R. Gromacs - a Message-Passing Parallel Molecular-Dynamics Implementation. *Comput. Phys. Commun.* **91,** 43-56 (1995).
6. Voss, N. R., Gerstein, M., Steitz, T. A., Moore, P. B. The geometry of the ribosomal polypeptide exit tunnel. *J. Mol. Biol.* **360,** 893-906 (2006).
7. James, F., Roos, M. Minuit - System for Function Minimization and Analysis of Parameter Errors and Correlations. *Comput. Phys. Commun.* **10,** 343-367 (1975).
8. Oang, K. Y., *et al.* Sub-100-ps structural dynamics of horse heart myoglobin probed by time-resolved X-ray solution scattering. *Chem. Phys.* **442,** 137-142 (2014).
