# Supplementary material for: Ultrafast coherent motion and helix rearrangement of homodimeric hemoglobin visualized with femtosecond X-ray solution scattering
Source: Nat Commun. 2021 Jun 16;12:3677. doi: 10.1038/s41467-021-23947-7 (PMC8209046; doi:10.1038/s41467-021-23947-7)
Supplement: Supplementary file 2 — Reporting summary [file 41467_2021_23947_MOESM2_ESM.pdf]

nature research

Corresponding author(s): Hyotcherl Ihee

Last updated by author(s): May 22, 2021

Reporting Summary

Nature Research wishes to improve the reproducibility of the work that we publish. This form provides structure for consistency and transparency in reporting. For further information on Nature Research policies, see our Editorial Policies and the Editorial Policy Checklist.

Statistics

For all statistical analyses, confirm that the following items are present in the figure legend, table legend, main text, or Methods section.

n/a	Confirmed	
☐	☒	The exact sample size (n) for each experimental group/condition, given as a discrete number and unit of measurement
☒	☐	A statement on whether measurements were taken from distinct samples or whether the same sample was measured repeatedly
☒	☐	The statistical test(s) used AND whether they are one- or two-sided<br>*Only common tests should be described solely by name; describe more complex techniques in the Methods section.*
☒	☐	A description of all covariates tested
☒	☐	A description of any assumptions or corrections, such as tests of normality and adjustment for multiple comparisons
☐	☒	A full description of the statistical parameters including central tendency (e.g. means) or other basic estimates (e.g. regression coefficient) AND variation (e.g. standard deviation) or associated estimates of uncertainty (e.g. confidence intervals)
☒	☐	For null hypothesis testing, the test statistic (e.g. F, t, r) with confidence intervals, effect sizes, degrees of freedom and P value noted<br>*Give P values as exact values whenever suitable.*
☐	☒	For Bayesian analysis, information on the choice of priors and Markov chain Monte Carlo settings
☒	☐	For hierarchical and complex designs, identification of the appropriate level for tests and full reporting of outcomes
☐	☒	Estimates of effect sizes (e.g. Cohen's d, Pearson's r), indicating how they were calculated

Our web collection on statistics for biologists contains articles on many of the points above.

Software and code

Policy information about availability of computer code

Data collection	Custom codes were used to collect the diffraction images and processing the images to generate the 1-D scattering curve and the difference scattering curves.
Data analysis	The FMINUIT package (version 2.3.1) which is developed based on the MINUIT package was used for fitting procedure. Custom codes were used to analyze for the kinetic and the structure refinement. For the theoretical scattering curve calculation, CRYSOL version 2.6 was used (J. Appl. Crystallogr. 28, 768-773 (1995)). To process the structure refinement results, GROMACS version 2016.5 was used. For the protein volume calculation, the volume calculation program package (version 1.2) developed and distributed by Voss et al (J. Mol. Biol. 360, 893-906 (2006)).

For manuscripts utilizing custom algorithms or software that are central to the research but not yet described in published literature, software must be made available to editors and reviewers. We strongly encourage code deposition in a community repository (e.g. GitHub). See the Nature Research guidelines for submitting code & software for further information.

Data

Policy information about availability of data

All manuscripts must include a data availability statement. This statement should provide the following information, where applicable:

- Accession codes, unique identifiers, or web links for publicly available datasets
- A list of figures that have associated raw data
- A description of any restrictions on data availability

PDB files with the PDB entries of 3sdh, 2grz, 5cmv, 5cnb, and 5cne were used for the analysis. Source data is provided. Other data that support the findings of this study are available from the corresponding author upon reasonable request.

Field-specific reporting

Please select the one below that is the best fit for your research. If you are not sure, read the appropriate sections before making your selection.

- [x] Life sciences
- [ ] Behavioural & social sciences
- [ ] Ecological, evolutionary & environmental sciences

For a reference copy of the document with all sections, see nature.com/documents/nr-reporting-summary-flat.pdf

Life sciences study design

All studies must disclose on these points even when the disclosure is negative.

Sample size	The sample size was not pre-determined using a statistical method. The sample size was determined based on previous studies using the same analysis methods.
Data exclusions	No candidate structures were excluded from the analysis.
Replication	Replication is not applicable to structure refinement due to the random nature of Monte-Carlo simulations.
Randomization	The candidate structures were generated from the randomly generated initial structures.
Blinding	Blinding is not applicable to our study, since there was no group allocation.

Reporting for specific materials, systems and methods

We require information from authors about some types of materials, experimental systems and methods used in many studies. Here, indicate whether each material, system or method listed is relevant to your study. If you are not sure if a list item applies to your research, read the appropriate section before selecting a response.

Materials & experimental systems

n/a	Involved in the study	
x		Antibodies
x		Eukaryotic cell lines
x		Palaeontology and archaeology
x		Animals and other organisms
x		Human research participants
x		Clinical data
x		Dual use research of concern

Methods

n/a	Involved in the study	
x		ChIP-seq
x		Flow cytometry
x		MRI-based neuroimaging
